# Synchronous profiling of mRNA N6-methyladenosine modifications and mRNA expression in high-grade serous ovarian cancer: a pilot study

**DOI:** 10.1038/s41598-024-60975-x

**Published:** 2024-05-07

**Authors:** Li Yang, Jingfang Liu, Yanlu Jin, Jie Xing, Jiejie Zhang, Xin Chen, Aijun Yu

**Affiliations:** 1 Department of Gynecological Oncology, Zhejiang Cancer Hospital, Hangzhou, 310022 Zhejiang China; 2 The Second Clinical Medical College, Wenzhou Medical University, Wenzhou, 325015 Zhejiang China

**Keywords:** High grade serous ovarian cancer, N6-Methyladenosine, Epitranscriptome, MeRIP-seq, RNA-seq, Cancer, Cell biology, Genetics, Oncology

## Abstract

This study aimed to synchronously determine epitranscriptome-wide RNA N6-methyladenosine (m6A) modifications and mRNA expression profile in high grade serous ovarian cancer (HGSOC). The methylated RNA immunoprecipitation sequencing (MeRIP-seq) was used to comprehensively examine the m6A modification profile and the RNA-sequencing (RNA-seq) was performed to analyze the mRNA expression profile in HGSOC and normal fallopian tube (FT) tissues. Go and KEGG analyses were carried out in the enrichment of those differentially methylated and expressed genes. MeRIP-seq data showed 53,794 m6A methylated peaks related to 19,938 genes in the HGSOC group and 51,818 m6A peaks representing 19,681 genes in the FT group. RNA-seq results revealed 2321 upregulated and 2486 downregulated genes in HGSOC. Conjoint analysis of MeRIP-seq and RNA-seq data identified differentially expressed genes in which 659 were hypermethylated (330 up- and 329 down-regulated) and 897 were hypomethylated (475 up- and 422 down-regulated). Functional enrichment analysis indicated that these differentially modulated genes are involved in pathways related to cancer development. Among methylation regulators, the m6A eraser (FTO) expression was significantly lower, but the m6A readers (IGF2BP2 and IGF2BP3) were higher in HGSOC, which was validated by the subsequent real-time PCR assay. Exploration through public databases further corroborated their possible clinical application of certain methylation regulators and differentially expressed genes. For the first time, our study screens the epitranscriptome-wide m6A modification and expression profiles of their modulated genes and signaling pathways in HGSOC. Our findings provide an alternative direction in exploring the molecular mechanisms of ovarian pathogenesis and potential biomarkers in the diagnosis and predicting the prognosis of the disease.

## Introduction

Ovarian cancer ranks as the third most common cancer and the deadliest gynecologic malignancy, with 313,959 new cases and 207,252 deaths worldwide in 2020^1^. High-grade serous ovarian cancer (HGSOC) is the most lethal subtype accounting for 60–80% of the disease^2,3^. Many clinical studies indicate that ovarian cancers originate in the fallopian tube (FT)^4–7^. Due to nonspecific and vague signs and symptoms and a lack of definitive screening tools, more than 70% of patients are diagnosed at an advanced stage (stage III/IV)^8^. Surgical debulking and chemotherapy are currently the main treatments. Almost all patients relapse within two years and most of them develop drug resistance. Despite the recent progress of surgical techniques and the adoption of PARP inhibitors, the overall survival of HGSOC patients has not been significantly improved. A better understanding of the molecular mechanism for HGSOC pathogenesis and progression is critical for developing new strategies for improved prevention, early detection, and treatment.

N6-methyladenosine (m6A) is the most prevalent mRNA modification in eukaryotic cells, which also dynamically and reversibly modifies transfer RNA, small nuclear RNA, circular RNA and long-chain non-coding RNA^9,10^. The m6A modification is catalyzed by the methyltransferases or writers, such as METTL3/14/16, RBM15/15B, ZC3H3, VIRMA, and KIAA1429, removed by the demethylases or erasers, including FTO and ALKBH5, and modulated by binding proteins or readers, such as YTHDF1/2/3, YTHDC1/2, IGF2BP1/2/3 and HNRNPA2B1^11–13^. The modification changes RNA secondary structures, splicing, translation, stability, and translocation, and participates in various biological processes and functions^14–16^. Aberrant m6A levels are associated with many pathological processes in the initiation and progression of varied types of human cancers^12,17^.

Some of the m6A regulators have been correlated with the occurrence and progression of ovarian cancer^9,12,13,18,19^. It is still unknown how the global m6A modifications and their modulated genes coordinately promote the development and progression of ovarian cancer. The methylated RNA immunoprecipitation sequencing (MeRIPseq) technology has become the powerful tool to examine an unbiased genome-wide m6A modification^20,21^. In this study, we applied MeRIP-seq to investigate the global m6A modification and RNA-sequencing (RNA-seq) to determine gene expression profile in HGSOC and normal fallopian tube (FT) tissues. Our findings would assist in exploring the molecular mechanism, diagnostic markers, and therapeutic targets for ovarian cancer^22^.

## Materials and methods

### Clinical specimens

Both HGSOC and normal FT tissues were collected at the time of surgery in Zhejiang cancer hospital in 2021. Normal FT tissues were obtained from patients with uterine leiomyomas. Samples were quickly snap-frozen in liquid nitrogen after surgical incision and then preserved at − 80 ℃ before use. None of these patients received chemotherapy or radiotherapy before the surgery. Two independent gynecological pathologists confirmed their diagnosis. A total of eleven HGSOC and eleven FT tissues were randomly selected for this study (Supplemental Table S1).

### Ethical approvement

The study was approved by the Institutional Review Board of Zhejiang cancer hospital. Each patient signed a written informed consent in the study before surgery. The study was performed in accordance with the Declaration of Helsinki.

### RNA extraction from HGSOC and normal FT tissues

Total RNA was isolated and purified using TRIzol reagent (Invitrogen, Carlsbad, CA, USA) following the manufacturer's procedure. The RNA amount and purity of each sample were quantified using NanoDrop ND-1000 (NanoDrop, Wilmington, DE, USA), and the RNA integrity was assessed by Bioanalyzer 2100 (Agilent, CA, USA). RNAs from three HGSOC and three normal FT tissues for the sequencing analyses.

### MeRIP-seq and RNA-seq in HGSOC and normal FT tissues

Poly (A) RNA was purified from 50 μg total RNA using Dynabeads Oligo (dT)25-61005 (Thermo Fisher, CA, USA) by two rounds of purification. Then the poly(A) RNA was fragmented into small pieces using Magnesium RNA Fragmentation Module (NEB, cat.e6150, USA) under 86 ℃ 7 min. The cleaved RNA fragments were incubated for 2 h at 4 ℃ with m6A-specific antibody (No. 202003, Synaptic Systems, Germany) in IP buffer (50 mM Tris–HCl, 750 mM NaCl and 0.5% Igepal CA-630). The IP RNA was reversely transcribed to cDNA by SuperScript™ II Reverse Transcriptase (Invitrogen, cat. 1896649, USA), which was next used to synthesize U-labeled second-stranded DNAs with E. coli DNA polymerase I (NEB, cat.m0209, USA), RNase H (NEB, cat. m0297, USA) and dUTP Solution (Thermo Fisher, cat.R0133, Waltham, MA, USA). An A-base was added to the blunt ends of each strand, preparing for ligation to the indexed adapters. Dual-index adapters were ligated to the fragments, and size selection was performed with AMPureXP beads. After the heat-labile UDG enzyme (NEB, cat. m0280, USA) treatment of the U-labeled second-stranded DNAs, the ligated products were amplified with PCR by the following conditions: initial denaturation at 95 ℃ for 3 min; 8 cycles of denaturation at 98 ℃ for 15 s, annealing at 60 ℃ for 15 s, and extension at 72 ℃ for 30 s; and then final extension at 72 ℃ for 5 min. At last, we performed paired-end sequencing (PE150) on an Illumina Novaseq™ 6000 platform (LC-Bio Technology CO., Ltd., Hangzhou, China) following the vendor's recommended protocol.

### Data analysis

Cutadapt and perl scripts in-house were used to remove reads that contained adaptor contamination, low quality bases, and undetermined base. The sequence quality was verified by Fastp. The high-quality clean IP and input reads were aligned to the genome sequences of homo sapiens (Version: v96) with default parameters using HISAT2 software. The aligned reads were used for peak calling of the MeRIP regions by R package exomePeak, while IGV software was used to visualize identified m6A peaks with bed or bam formats (http://www.igv.org/). MEME and HOMER were used for de novo and known motif finding, followed by localization of the motif involving peak summit by perl scripts in-house. ChIPseeker was used to conduct called peaks annotation and intersection with gene architecture.

The levels of methylated peaks were normalized by RPM (Reads per million reads). The expression levels for all genes from input libraries were calculated as FPKM (Fragments per kilobase of exon per million fragments mapped = total_exon_fragments/mapped_reads (millions) X exon_length (kB)) using StringTie. Based on the log2 (fold change) > 1 or log2 (fold change) < − 1, the differentially expressed methylation peaks were identified by the Poisson Test (P < 0.05), and their related genes by the Exact test (P < 0.05). The levels of mRNAs in RNA-seq data were presented as FPKM. The differentially expressed mRNAs were identified by the same fold change criteria and the Exact test (P < 0.05) using R package edgeR. Go and KEGG^23^ analyses were performed in differentially methylated and expressed genes.

### Real-time (RT)-PCR validation of m6A regulator genes

The analyses were performed in 11 HGSOC and 11 FT tissues (Supplementary Table S1). The primer of the target genes and β-actin in RT-qPCR were designed and synthesized by Sangon Biotech Co., Ltd (Shanghai, China) (Supplementary Table S2). The SuperScript IV cDNA synthesis Kit (Thermo Fisher, Waltham, MA, USA) was used for cDNA synthesis. SYBR Green master mix from Bimake (Munich, German) and the ABI 7500 system (Thermo Fisher, Waltham, MA, USA) was used for RT-PCR. All PCR experiments were conducted in triplicate and the average value was calculated. The gene expression values were normalized to β-actin.

### Immunohistochemical (IHC) staining of m6A regulator proteins

The protein expression of expressed m6A regulators was examined by IHC staining in tissues of HGSOC (n = 10) and normal FT (n = 4). The paraffin embedded tissues were sectioned at 4 µm. After deparaffinization and rehydration, the tumor tissue sections were first processed with antigen retrieval, and then sequentially blocked with freshly prepared 3% hydrogen peroxide and 5% bovine serum albumin (BSA). Primary antibodies (1:300, ABCAM and Proteintech, Shanghai, China) were incubated overnight at 4 °C. These antibodies included FTO (ab280081), YTHDF1 (ab230330), YTHDF2 (ab220163), WTAP (ab195380), IGF2BP2 (ab129071), METTL3 (ab195352), METTL14 (ab300104), ALKBH5 (ab195377), and IGF2BP3 (Proteintech 81805-1-RR). After treatment with corresponding secondary antibodies (1:100, ABCAM), the section was stained with 3,3′-Diaminobenzidine (DAB) for 5–10 min and then counterstained with hematoxylin counterstaining.

The intensity and distribution patterns of IHC staining were evaluated using a semi-quantitative Immunoreactive Score (IRS). The IRS is calculated based on two parameters: the staining intensity (0, no staining; 1, weak staining; 2, moderate staining; and 3, strong staining) and the percentage of positive cells (0, no positive cells;1, less than 10% of cells positive; 2, 10–50% of cells positive; 3, 51–80% of cells positive; 4, more than 80% of cells positive). The IRS was calculated by multiplying these two scores.

### Correlation of gene expression with clinical significance using public databases

We then explored the expression of genes between ovarian cancer and normal ovary tissues using the TCGA and GTEx public databases (https://xenabrowser.net)^24^. The Kaplan–Meier Plotter database was applied to examine the association between the expression of genes and survival in serous ovarian cancer patients (https://kmplot.com/analysis/)^25^.

### Statistical analysis

The Mann–Whitney U Test was performed to compare gene expression from the RT-PCR, and the different IRS scores of IHC staining between HGSOC and FT groups. Statistical analyses were performed using SPSS (version 22.0, SPSS Inc., Chicago, IL, USA). A P value less than 0.05 was considered statistically significant.

## Results

### Global m6A modification patterns in HGSOC and FT

**Figure 1:**
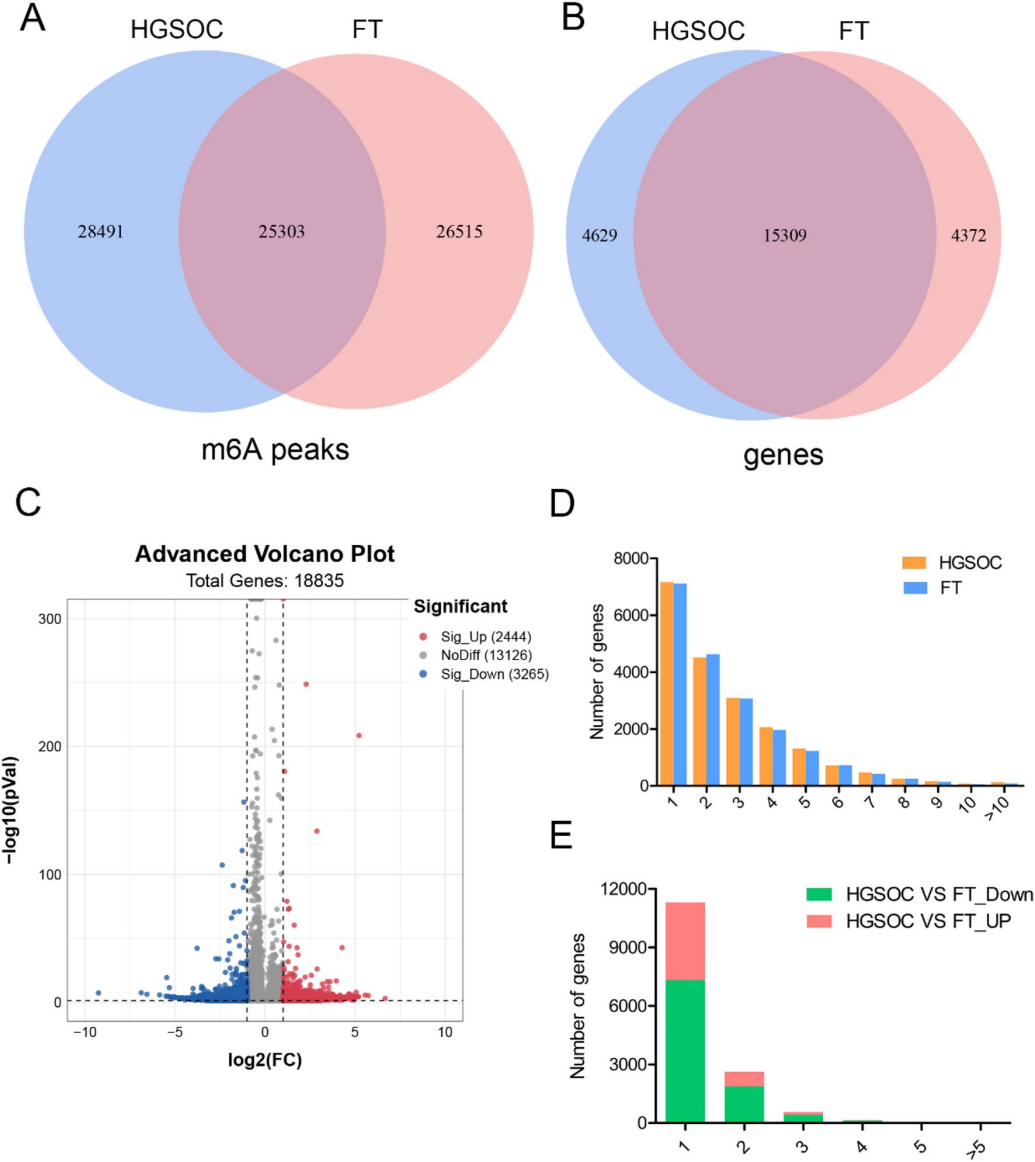
Overview of m6A distributions in genes between HGSOC and FT tissues (n = 3 each). Venn diagrams showing (**A**) m6A methylation peaks and (**B**) their related genes only in HGSOC, or only in FT tissues, or in both. (**C**) Volcano plots of the significantly differentially m6A methylated genes between HGSOC and FT tissues. (**D**) Number of m6A peaks per gene in HGSOC and FT tissues. (**E**) Number of m6A peaks per differentially methylated gene. FT, fallopian tube; HGSOC, high grade serous ovarian cancer.

Reads data and quality testing for MeRIP-seq were shown in Supplementary Table S3. Venn diagrams were constructed to show the m6A peaks (Fig. 1A) and their modified genes (Fig. 1B) in the two groups. A total of 28,491 m6A peaks (Fig. 1A) and their modified 4629 genes (Fig. 1B) were detected in only the HGSOC group. As many as 26,515 m6A peaks (Fig. 1A) and their modified 4372 genes (Fig. 1B) were identified only in the FT group. In addition, 25,303 m6A peaks and their modified 15,309 genes were shared by both groups (Fig. 1A,B).

The abundance of m6A methylated genes between the two groups was compared. A total of 2444 genes were hyper-methylated and 3265 genes were hypo-methylated in the HGSOC group compared with the FT group (Fig. 1C). The top 30 methylated genes with altered m6A methylated peaks are listed in Table 1. There was no significant difference in the number of m6A peaks per gene between the two groups (Fig. 1D). The majority of genes (75%) had 1–3 m6A peaks and about 36% of genes had one modified m6A peak (Supplementary Fig. 1A). Among those differentially m6A modified genes, 77% of them harbored one m6A methylated peak and approximately 98% of genes had 1–3 m6A modified sites (Fig. 1E and Supplementary Fig. 1B).

**Table 1:** The top 30 differentially methylated genes between HGSOC and FT.

Gene name	Gene ID	Fold change (Log2)	M6A methylation	Chromosome	Peak start	Peak end	Adjusted P value
ARPP21	ENSG00000172995	6.67	Up	chr3	35639801	35639901	0.007
ZNF560	ENSG00000198028	5.74	Up	chr19	9466354	9467254	< 0.001
CBS	ENSG00000160200	5.56	Up	chr21	43053740	43055203	< 0.001
FP236383	ENSG00000280614	5.22	Up	chr21	8393418	8393968	< 0.001
ITGB7	ENSG00000139626	5.18	Up	chr12	53200261	53200411	< 0.001
FAM240C	ENSG00000216921	5.18	Up	chr2	241894034	241894334	0.001
ARID4B	ENSG00000054267	5.07	Up	chr1	235327811	235328111	< 0.001
CBS	ENSG00000160200	5.06	Up	chr21	43056003	43058968	< 0.001
IGHV3-74	ENSG00000224650	5.04	Up	chr14	106810441	106810894	< 0.001
AL390860	ENSG00000235749	4.99	Up	chr1	247639954	247640079	0.001
AL929601	ENSG00000277367	4.93	Up	chr14	18889358	18889508	0.046
SAMMSON	ENSG00000240405	4.93	Up	chr3	70014419	70014594	0.046
ANKRD22	ENSG00000152766	4.91	Up	chr10	88823481	88831964	0.001
ALDH3B1	ENSG00000006534	4.90	Up	chr11	68016375	68016550	0.001
SIK1B	ENSG00000275993	4.89	Up	chr21	6116982	6118271	< 0.001
AL023754	ENSG00000224260	− 9.25	Down	chr1	209528554	209528654	< 0.001
RNF165	ENSG00000141622	− 5.51	Down	chr18	46461421	46461521	< 0.001
AC022414	ENSG00000284762	− 5.12	Down	chr5	77140607	77140957	0.001
RERG	ENSG00000134533	− 4.37	Down	chr12	15221341	15337450	0.004
ADH6	ENSG00000172955	− 4.31	Down	chr4	99204387	99204562	0.005
LRRC34	ENSG00000171757	− 4.00	Down	chr3	169796742	169796892	0.009
CDH26	ENSG00000124215	− 6.56	Down	chr20	60031425	60033609	0.000
LINC01320	ENSG00000228262	− 4.12	Down	chr2	34735613	34737572	0.006
EBF2	ENSG00000221818	− 3.60	Down	chr8	25842099	25842224	0.026
PTGS2	ENSG00000073756	− 3.64	Down	chr1	186674040	186674140	0.022
RABGAP1L	ENSG00000152061	− 4.07	Down	chr1	174246238	174250541	0.008
PDE10A	ENSG00000112541	− 4.12	Down	chr6	165327361	165327486	0.008
C11orf16	ENSG00000176029	− 4.39	Down	chr11	8929117	8929217	0.005
FRRS1L	ENSG00000260230	− 4.25	Down	chr9	109132817	109132917	0.006
MYOCD	ENSG00000141052	− 5.06	Down	chr17	12766399	12767524	0.001

FT, fallopian tube; HGSOC, high grade serous ovarian cancer.

### M6A topological patterns in HGSOC

**Figure 2:**
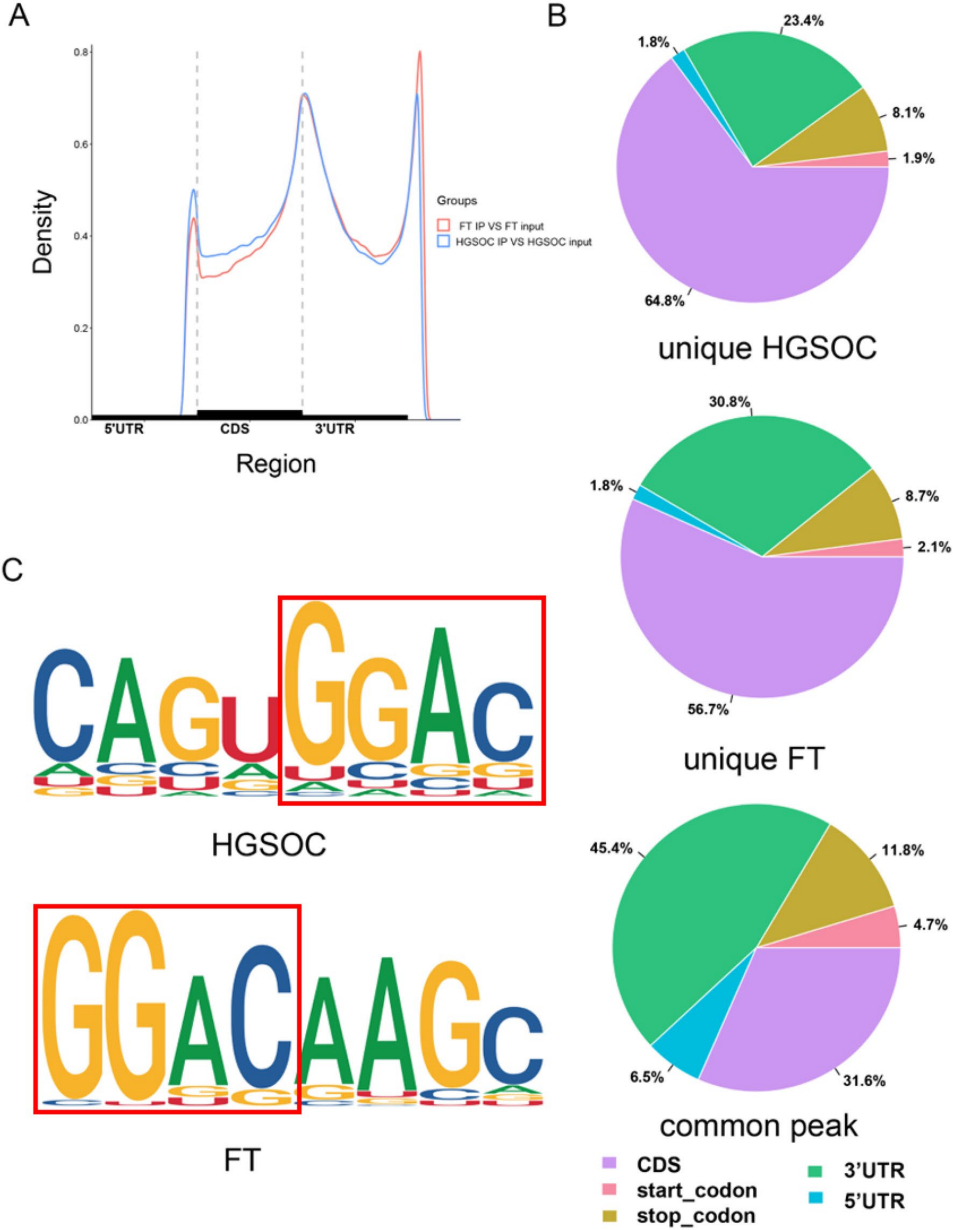
Topological patterns of m6A distributions in genes between HGSOC and FT tissues (n = 3 each). (**A**) Distribution site of the m6A peaks on all the transcripts in HGSOC and FT. Each transcript was divided into 5ʹ-UTR, CDS, and 3ʹ-UTR regions. (**B**) Pie charts showed the distribution proportion of unique and common m6A peaks between HGSOC and FT groups. (**C**) The representative m6A motifs displayed GGAC conserved sequence from altered m6A peaks in HGSOC and FT groups. CDS, coding sequence; FT, fallopian tube; HGSOC, high grade serous ovarian cancer; UTR, untranslated region.

The distribution of m6A peaks in the entire epitranscriptome was analyzed in both HGSOC and FT groups. The m6A peaks were identified in the start and codons, 3′ untranslated regions (3′UTR), 5′UTR, and coding sequence (CDS) on transcripts. In general, these m6A peaks were more enriched in CDS and 3′UTR (Fig. 2A). The distributions of differential and common m6A peaks between the two groups were displayed using pie graphs (Fig. 2B). The m6A hypermethylated peaks in the HGSOC group were found more in CDS (64.8% vs 57.6%) but less in 3′UTR (23.4% vs 30.8%) compared to those in the FT group. The common m6A peaks showed a relatively higher proportion in 3′UTR than in CDS (45.4% vs 31.6%). The classical consensus GGAC motif structures were identified and thus confirmed the authenticity of m6A peaks in both HGSOC and FT groups (Fig. 2C).

### Differentially methylated m6A genes were enriched in important signaling pathways

**Figure 3:**
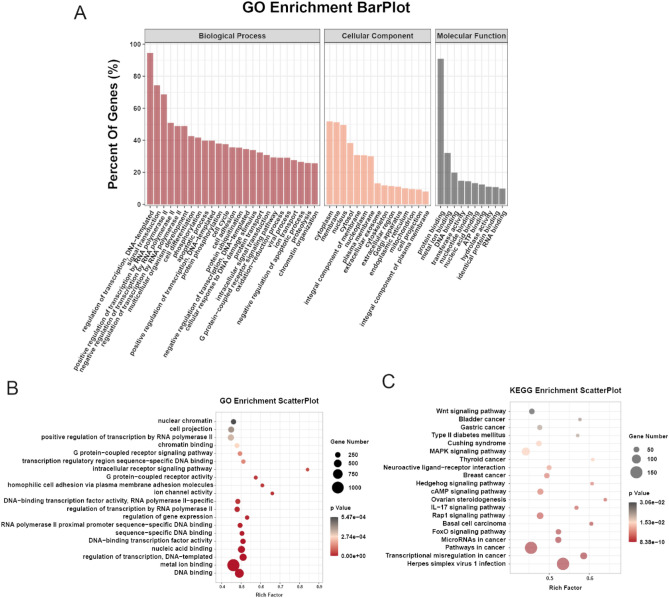
Result of GO function and KEGG pathway analyses in differentially methylated genes. (**A**) Bar plot of the top 25 biological process terms, 15 cellular component terms and 10 molecular function terms of differentially methylated genes in HGSOC in GO analysis. (**B**) Scatter plotter of the top 20 significant GO terms. (**C**) A scatter plot of the top 20 significant KEGG enrichment pathways.

Gene ontology (GO) and Kyoto encyclopedia of genes and genomes (KEGG) analyses were performed in differentially methylated genes to investigate the biological significance and potential functions of m6A modification. In GO analysis, genes were classified into three functional domains: biological process, cellular component, and molecular functions. The top 25 biological process terms, 15 cellular component terms and 10 molecular function terms are listed in Fig. 3A. The differentially methylated genes were mainly enriched in the regulation of transcription, signal transduction, and regulation of transcription by RNA polymerase II, et al. In terms of cellular components, genes were mainly involved in cell cytoplasm, membrane, and nucleus, et al. In molecular function terms, genes played a part in protein binding, metal ion binding, and DNA binding, et al. GO enrichment analysis revealed that genes were significantly enriched in DNA binding, metal ion binding, regulation of transcription, G protein-coupled receptor signaling pathway, et al. (Fig. 3B). KEGG pathway analysis revealed that transcriptional misregulation, FoxO signaling pathway, Rap1 signaling pathway, IL-17 signaling pathway, cAMP signaling pathway, Hedgehog signaling pathway, MAPK signaling pathway and Wnt signaling pathways were significantly altered in HGSOC (Fig. 3C).

### Functional analysis of differentially expressed genes between HGSOC and FT tissues

**Figure 4:**
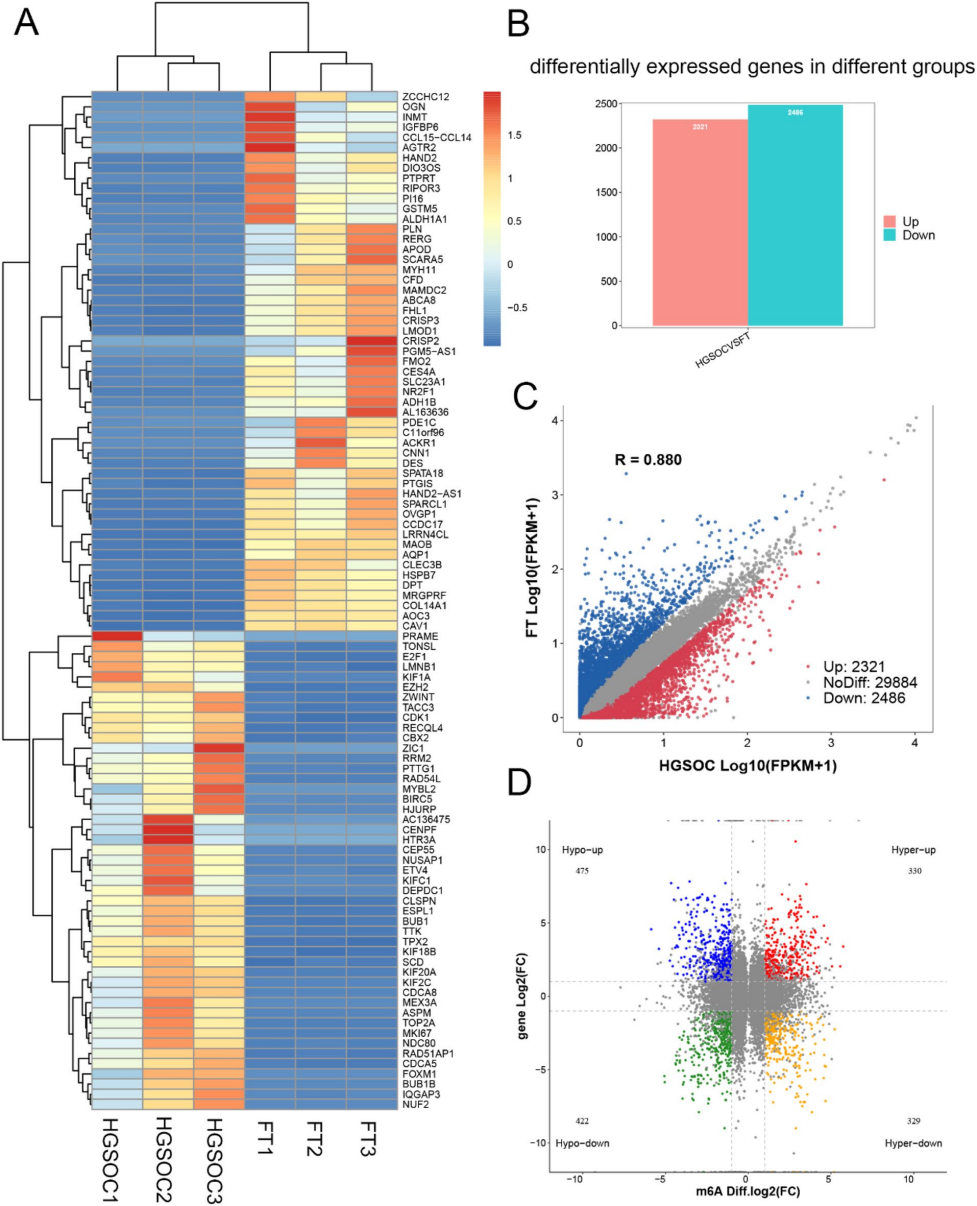
Conjoint analysis result of MeRIP-seq and RNA-seq data. (**A**) Heatmap plots of the differentially expressed genes between HGSOC and FT tissues. (**B**) The number of differentially expressed genes. (**C**) Scatter plot of the differentially expressed genes. (**D**) The four-quadrant plot of differentially m6A methylated and expressed genes. FT, fallopian tube; HGSOC, high grade serous ovarian cancer.

The RNA-seq was used to examine differentially expressed genes between HGSOC and FT tissues. The heatmap was used to present the top differentially expressed genes between the two groups (Fig. 4A). There were 4807 differentially expressed genes between two groups, including 2321 upregulated and 2486 downregulated in HGSOC. The expression of 29,884 genes was not significantly different between groups (Fig. 4B,C).

### Conjoint analysis of m6A methylation and gene expression profiles

**Figure 5:**
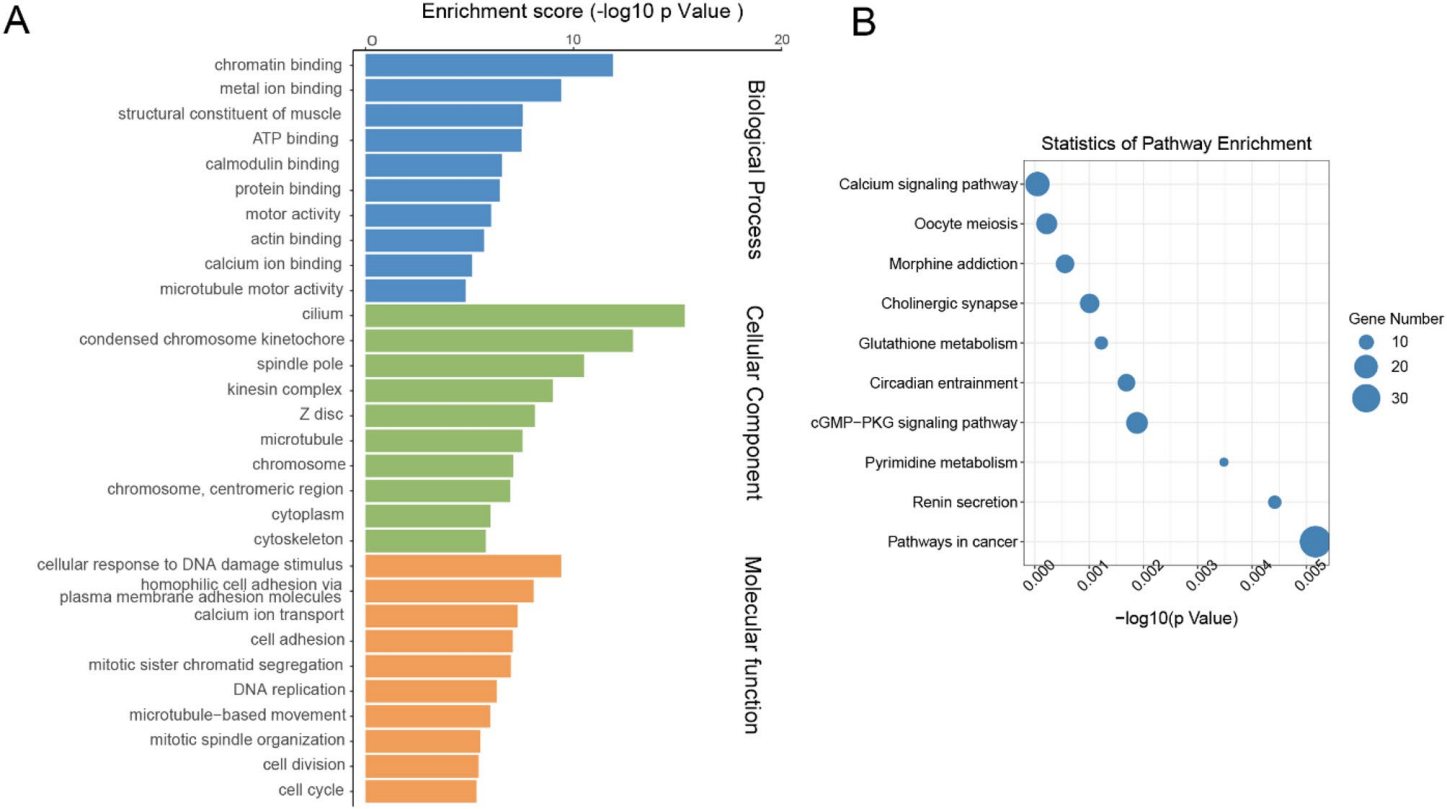
Result of GO terms and KEGG pathways of differentially methylated and expressed genes between HGSOC and FT tissues (n = 3 each). (**A**) Bar plot of the top 10 biological processes, cellular component and molecular function terms in GO analysis. (**B**) Scatter plot of the top 10 KEGG pathways. FT, fallopian tube; HGSOC, high grade serous ovarian cancer.

By conjoint analysis of MeRIP-seq and RNA-seq data, genes were classified into four groups: 659 hyper-methylated m6A peaks with 330 genes upregulated (hyper-up) and 329 genes downregulated (hyper-down), and 897 hypo-methylated m6A peaks with 475 genes upregulated (hypo-up), and 422 genes downregulated (hypo-down) (Fig. 4D). Results of GO and KEGG analyses in these four groups of genes are presented in Supplementary Fig. S2A–H. The top differentially modulated genes in each group are presented in Tables 2 and 3. We performed GO and KEGG analyses in those differentially m6A methylated and expressed genes. The top 10 terms of biological process, cellular component and molecular functions are listed in Fig. 5A. KEGG pathway analysis revealed that these genes were enriched in cGMP-PKG pathway and calcium signaling pathway, et al. (Fig. 5B).

**Table 2:** The top hypermethylated and differentially expressed genes in HGSOC vs FT.

Gene name	Gene ID	Fold change (Log2)	Gene expression	M6a	Chromosome	Peak start	Peak end	Adjusted P value
MYH11	ENSG00000133392	− 9.00	Down	Up	chr16	15724335	15725060	< 0.001
MYBPC1	ENSG00000196091	− 7.90	Down	Up	chr12	101685673	101685823	< 0.001
CNN1	ENSG00000130176	− 7.74	Down	Up	chr19	11550086	11550323	< 0.001
CBLN4	ENSG00000054803	− 7.49	Down	Up	chr20	55997356	55997756	< 0.001
TCTEX1D1	ENSG00000152760	− 7.23	Down	Up	chr1	66777587	66778712	< 0.001
PDE1C	ENSG00000154678	− 7.21	Down	Up	chr7	31789484	31790234	< 0.001
PDE1C	ENSG00000154678	− 7.21	Down	Up	chr7	31788059	31788384	< 0.001
PDE1C	ENSG00000154678	− 7.21	Down	Up	chr7	31753453	31783859	< 0.001
AL078622	ENSG00000286381	− 7.14	Down	UP	chr22	49047483	49048458	< 0.001
ALDH1A2	ENSG00000128918	− 6.89	Down	Up	chr15	57990266	57990566	< 0.001
MIR1-1HG-AS1	ENSG00000174403	− 6.82	Down	Up	chr20	62544342	62544817	< 0.001
LINC01550	ENSG00000246223	− 6.69	Down	UP	chr14	97969457	97978011	< 0.001
CLSPN	ENSG00000092853	6.23	Up	Up	chr1	35734648	35735198	< 0.001
CENPF	ENSG00000117724	6.27	Up	Up	chr1	214641318	214653590	< 0.001
HJURP	ENSG00000123485	6.32	Up	Up	chr2	233837208	233845732	< 0.001
LRP2	ENSG00000081479	6.47	Up	Up	chr2	169127108	169127433	< 0.001
FBN3	ENSG00000142449	6.61	Up	Up	chr19	8065401	8065651	< 0.001
FBN3	ENSG00000142449	6.61	Up	Up	chr19	8065851	8066251	< 0.001
MYBL2	ENSG00000101057	6.83	Up	Up	chr20	43687032	43702673	< 0.001
RNF144A-AS1	ENSG00000228203	6.94	Up	Up	chr2	6915267	6916817	< 0.001

FT, fallopian tube; HGSOC, high grade serous ovarian cancer.

**Table 3:** The top hypomethylated and differentially expressed genes in HGSOC vs FT.

Gene name	Gene ID	Fold change (Log2)	Gene expression	M6A	Chromosome	Peak start	Peak end	Adjusted P value
MYH11	ENSG00000133392	− 9.00	Down	Down	chr16	15703134	15704109	< 0.001
MYH11	ENSG00000133392	− 9.00	Down	Down	chr16	15740136	15741560	< 0.001
PI16	ENSG00000164530	− 7.90	Down	Down	chr6	36964557	36964832	< 0.001
ALDH1A2	ENSG00000128918	− 6.89	Down	Down	chr15	57953423	57953648	< 0.001
TMED6	ENSG00000157315	− 6.64	Down	Down	chr16	69343249	69343640	< 0.001
NNAT	ENSG00000053438	− 6.52	Down	Down	chr20	37522986	37523236	< 0.001
PGM5	ENSG00000154330	− 5.90	Down	Down	chr9	68530692	68531061	< 0.001
PODN	ENSG00000174348	− 5.71	Down	Down	chr1	53063437	53066839	< 0.001
PODN	ENSG00000174348	− 5.71	Down	Down	chr1	53084577	53084752	< 0.001
SPARCL1	ENSG00000152583	− 5.66	Down	Down	chr4	87490859	87529498	< 0.001
SPARCL1	ENSG00000152583	− 5.66	Down	Down	chr4	87473334	87473559	< 0.001
KCNB1	ENSG00000158445	− 5.66	Down	Down	chr20	49310622	49364074	0.008
F10	ENSG00000126218	− 5.65	Down	Down	chr13	113140779	113141029	< 0.001
DTL	ENSG00000143476	5.58	Up	Down	chr1	212101004	212103286	< 0.001
COL9A1	ENSG00000112280	5.75	Up	Down	chr6	70215985	70216385	< 0.001
COL9A1	ENSG00000112280	5.75	Up	Down	chr6	70216460	70216685	< 0.001
AQP6	ENSG00000086159	5.84	Up	Down	chr12	49976935	49977135	< 0.001
AQP6	ENSG00000086159	5.84	Up	Down	chr12	49976610	49976710	< 0.001
TICRR	ENSG00000140534	5.94	Up	Down	chr15	89624917	89627435	< 0.001
RRM2	ENSG00000171848	6.04	Up	Down	chr2	10198798	10198973	< 0.001
RRM2	ENSG00000171848	6.04	Up	Down	chr2	10129571	10130046	< 0.001
GUCY1B2	ENSG00000123201	6.23	Up	Down	chr13	51004451	51006849	0.001
GUCY1B2	ENSG00000123201	6.23	Up	Down	chr13	51004276	51004401	0.001

FT, fallopian tube; HGSOC, high grade serous ovarian cancer.

### Expression of genes related to m6A regulators between HGSOC and FT tissues

**Figure 6:**
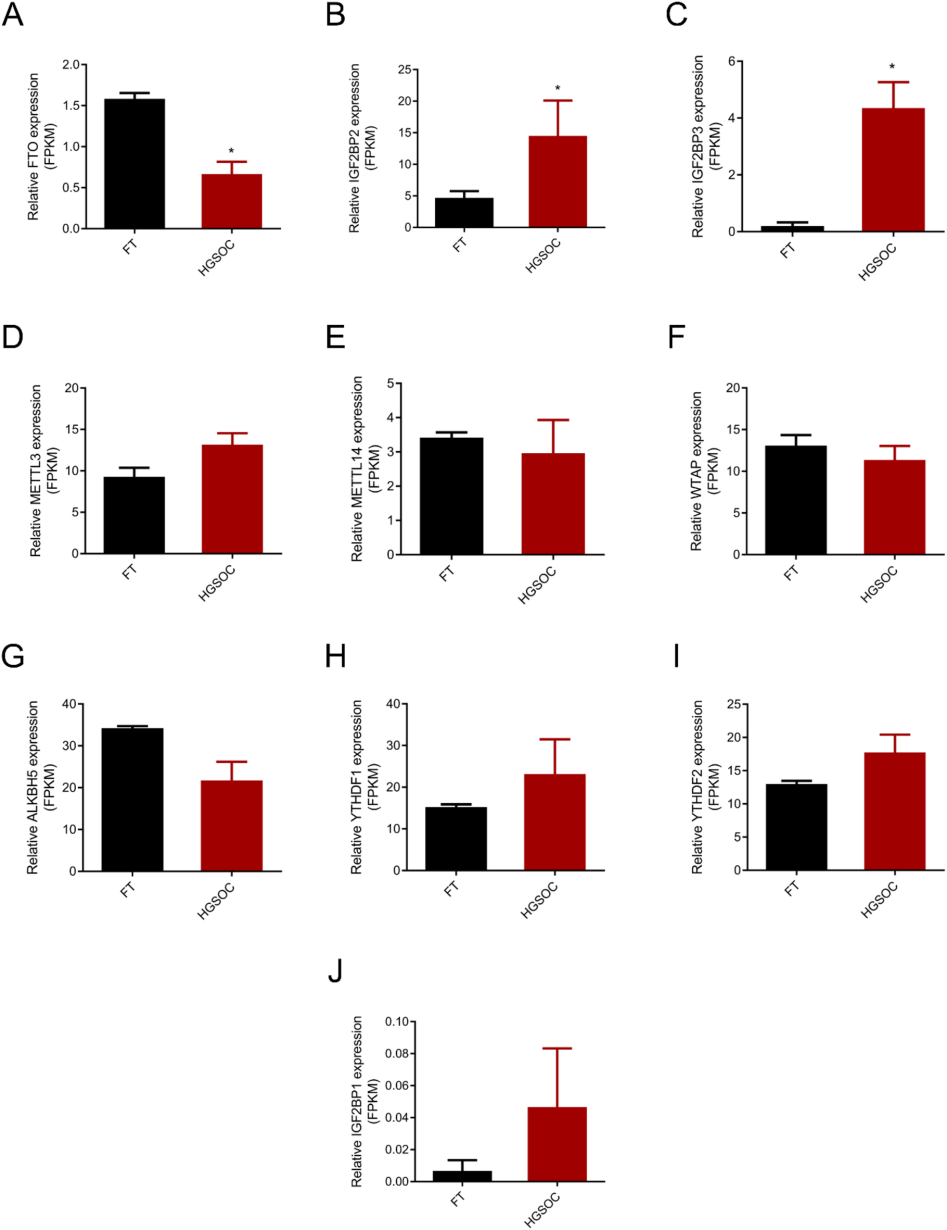
RNA-seq result of differently expressed genes in HGSOC and FT tissues (n = 3 each). FPKM, Fragments Per Kilobase Million; FT, fallopian tube; HGSOC, high grade serous ovarian cancer. *P value < 0.05.

**Figure 7:**
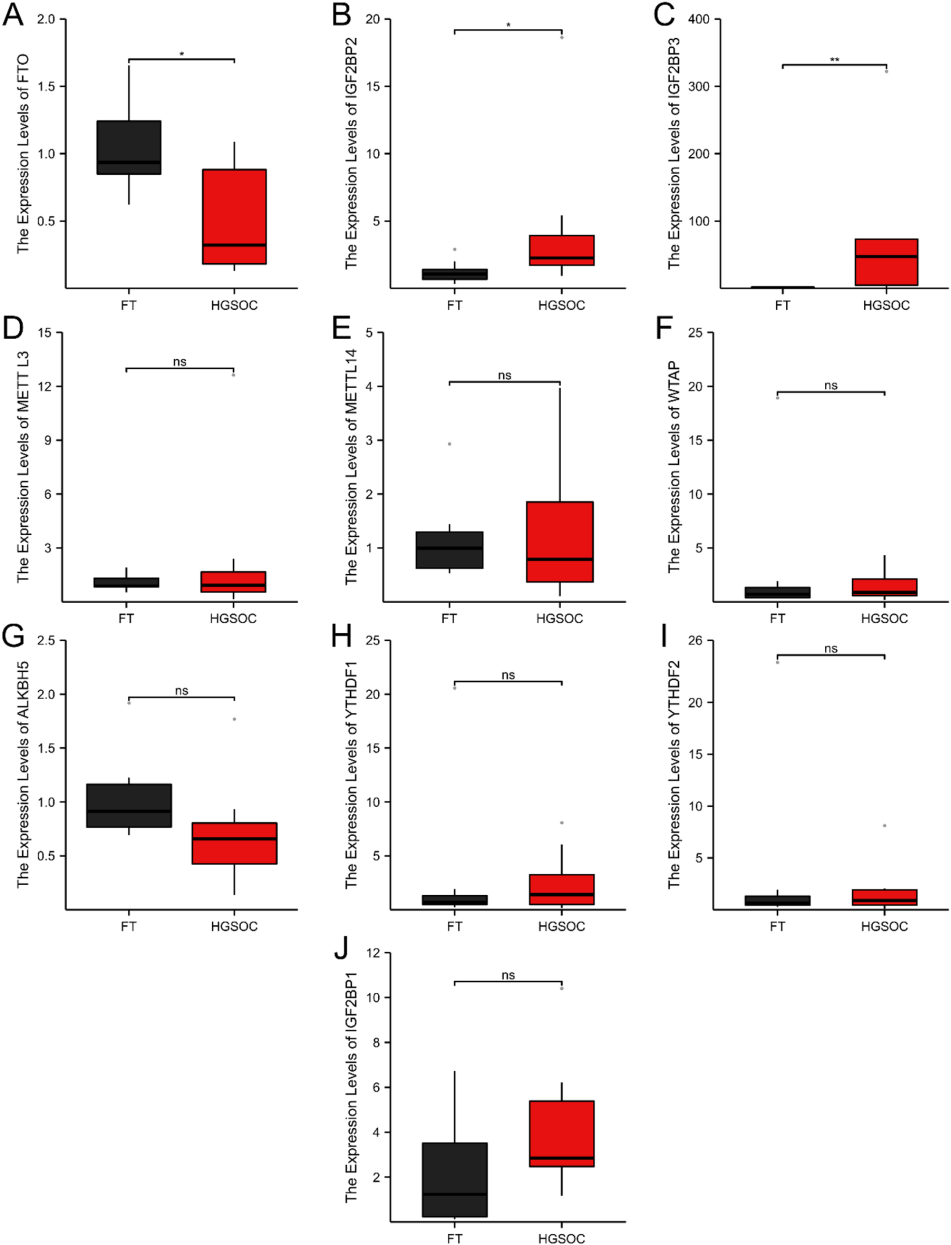
RT-PCR result of m6A methylation regulators in HGSOC (n = 11) and FT tissues (n = 11). FT, fallopian tube; HGSOC, high grade serous ovarian cancer; ns, not significant. *P value < 0.05, and **P value < 0.01.

RNA-seq data revealed the expression levels of genes related to m6A regulators in HGSOC and FT tissues (Fig. 6A–J). Our data showed that IGF2BP2 and IGF2BP3 were significantly increased, whereas FTO was significantly decreased in HGSOC. In contrast, there was no significant change in other m6A regulators. Expression of these genes was further examined by RT-PCT in 11 HGSOC and 11 FT tissues (Fig. 7A–J). The protein levels of these m6A regulators were further examined using IHC staining (Supplementary Fig. S3A,B). Both PCR and IHC staining results matched the expression patterns observed from profiling analyses.

### Results of m6A methylation regulators and differentially expressed genes in public databases

**Figure 8:**
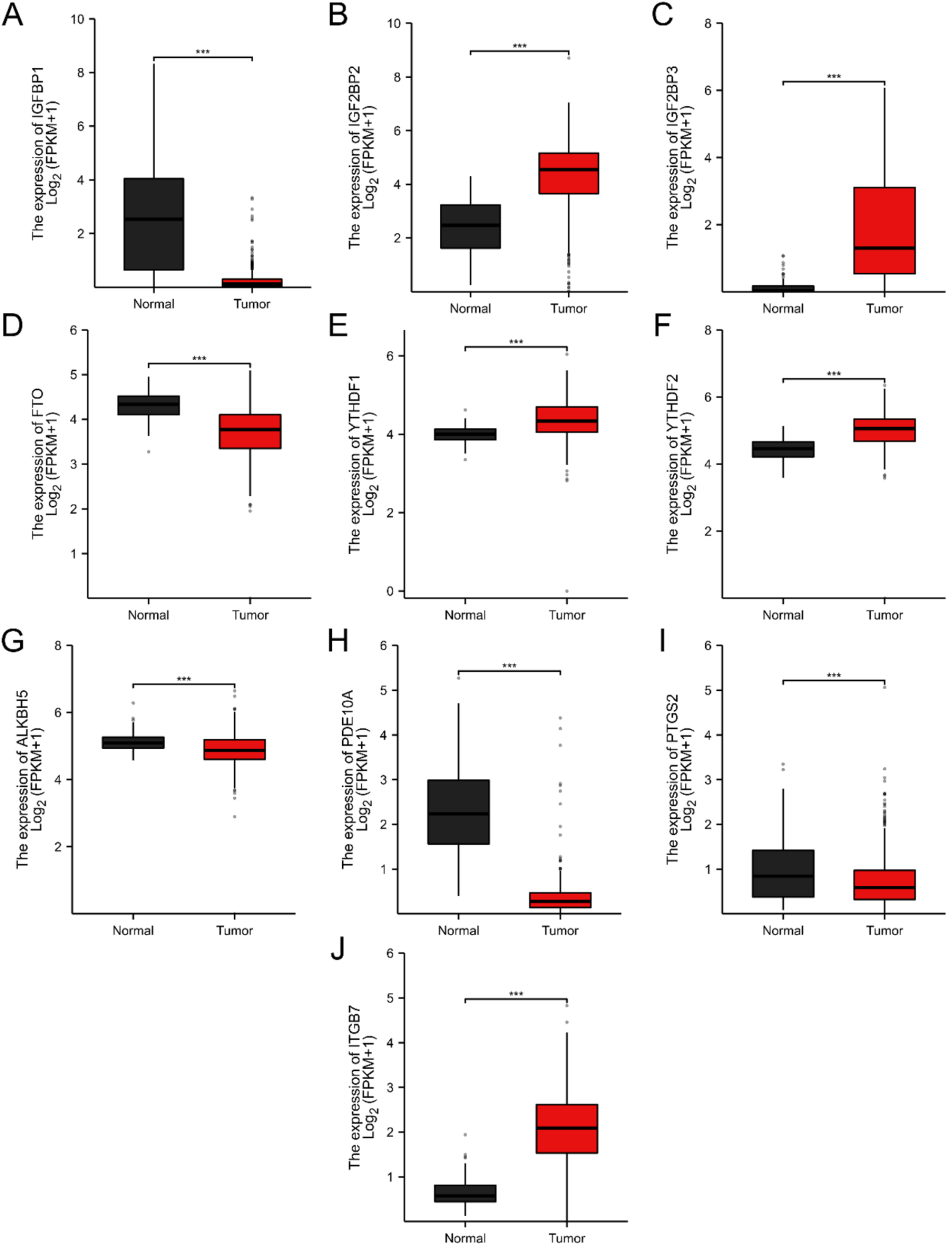
Levels of differently expressed genes in ovarian cancer (n = 427) and normal ovarian tissues (n = 88) using the TCGA and GTEx databases. *P value < 0.05 and **P value < 0.01. FT, fallopian tube; HGSOC, high grade serous ovarian cancer; ns, not significant; TPM, Transcripts Per Kilobase Million.

**Figure 9:**
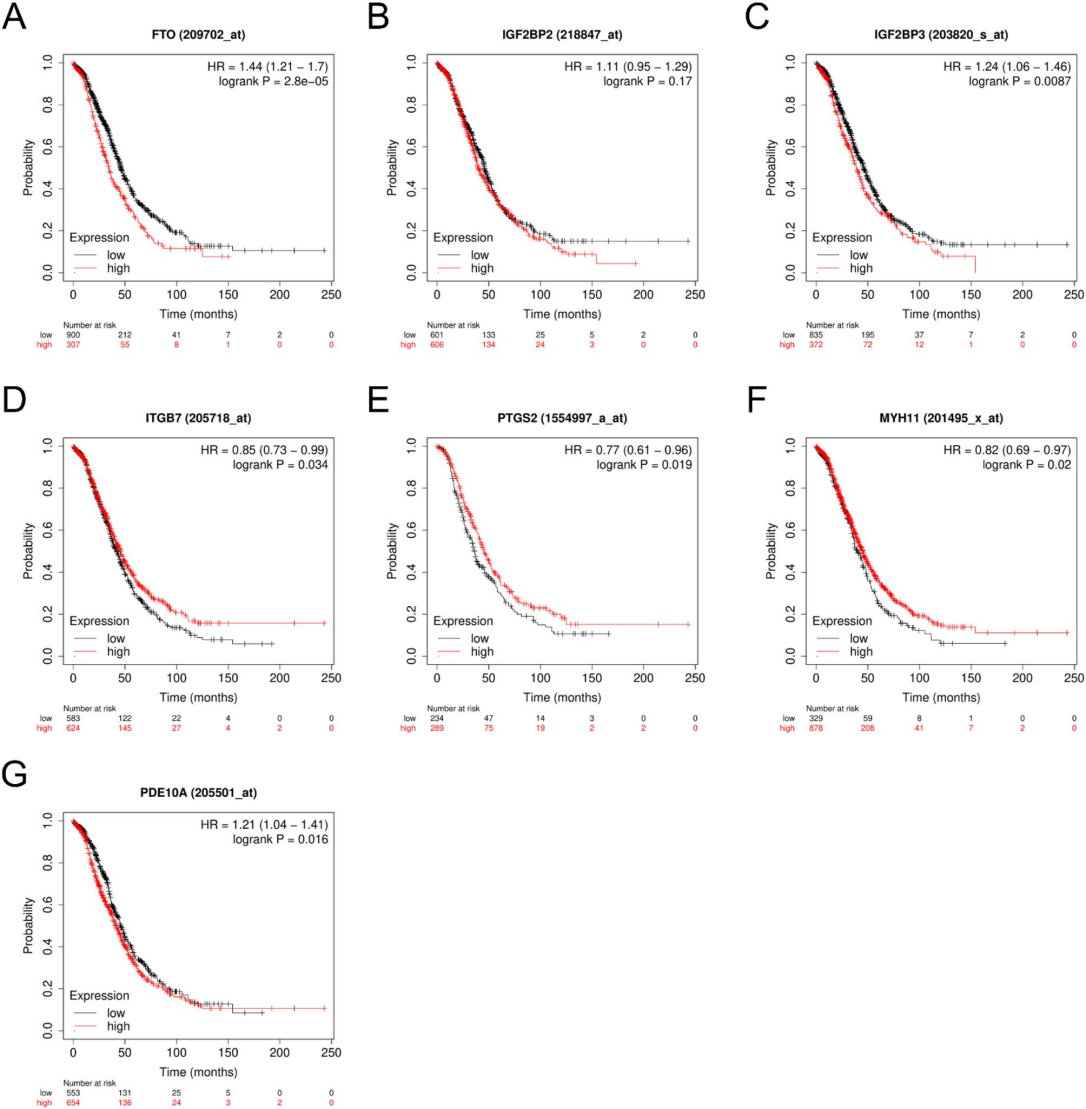
Survival curves of ovarian cancer patients with high or low expression of genes using the Kaplan Meier plotter database. HR, Hazard ratio.

We also explore differentially expressed genes in the TCGA and GTEx public databases. Consistent with our results, IGF2BP2 and IGF2BP3 were significantly increased in ovarian cancer, whereas FTO was significantly decreased in ovarian cancer. PDE10A and PTGS2 were significantly decreased, but ITGB7 was significantly increased in ovarian cancer (Fig. 8A–K). Data from the Kaplan–Meier Plotter database indicated the association between the expression of genes and survival in ovarian cancer patients (Fig. 9A–G).

## Discussion

Abnormal m6A modifications have been associated with proliferation, drug resistance, and prognosis of ovarian and other cancers^22,26,27^. The global m6A profile has previously been screened in several cancers, such as endometrioid ovarian cancer^28^, invasive malignant pleomorphic adenoma^29^, and colorectal cancer^30^. For the first time, this study examined the epitranscriptome-wide m6A methylation and synchronously determined the gene expression profile in HGSOC and FT tissues. Besides the feature of m6A modification sites, our study revealed the differential global m6A methylation and differentially expressed gene expression profile between HGSOC and FT tissues. The conjoint analysis further identified those differentially m6A modified and expressed genes. Enrichment analysis suggests the involvement of these genes in multiple complex functions and signaling pathways correlated with ovarian development and progression. Among them, m6A eraser FTO and readers IGF2BP2 and IGF2BP3 were differentially modulated between HGSOC and FT tissues. Parts of our findings were validated with subsequent RT-PCR and IHC staining. The expression levels and clinical roles of significant genes were further examined in ovarian cancer patients using public databases.

Previous studies have demonstrated the critical role of M6A regulators FTO, IGF2BP2 and IGF2BP3 in ovarian and other cancers. IGF2BP2 and IGF2BP3 were positively associated with the expression of C3AR1, a member of the G protein coupled receptor 1 family, in ovarian cancer^31^. C3AR1 overexpression significantly increased the proliferation of ovarian cancer SKOV3 cells and was associated with immunosuppression and poor prognosis of ovarian cancer^31^. Overexpression of C3AR1 was associated with a poor prognosis in patients with gastric adenocarcinoma^32^. IGF2BP2 contributed to colorectal cancer pathogenesis and progression by SOX2 m6A methylation and preventing its degradation^33^. IGF2BP3 was shown to modulate cell cycle, cell proliferation and angiogenesis through m6A modification to increase both expression and stability of cyclin D1 (CCND1) and VEGF mRNAs in colon cancer^34^. Downregulation of m^6^A erasers FTO and ALKBH5 increased FZD10 m^6^A modification and its mRNA expression, and reduced PARPi sensitivity in BRCA-mutated epithelial ovarian cancers^35^. Overexpression of FTO led to hypomethylation and reduced mRNA stability of two phosphodiesterase genes (PDE1C and PDE4B) but augmented cAMP signaling and dampen stemness features of ovarian cancer cells^19^. FTO decreased the m6A level and stability of SNAI1 mRNA, causing IGF2BP2 dependent downregulation of SNAI1 and inhibiting epithelial-mesenchymal transition^36^. Overexpression of FTO inhibited tumor growth in nude mice by facilitating the oxidative stress response and apoptosis of ovarian cancer cells via activation of the P53 signaling pathway^37^. Our study found that m6A eraser FTO was significantly lower, whereas IGF2BP2 and IGF2BP3 were significantly higher in HGSOC than in normal FT. The profiling data was confirmed by subsequent RT-PCR analyses in more samples. This change pattern of these genes between normal and ovarian cancer tissues is observed in data from the TCGA database. Data from public database Kaplan–Meier Plotter revealed that the expression of FTO and IGF2BF3 was significantly correlated with the prognosis of ovarian cancer patients.

Other m6A regulators, such as m6A writers METTL3, METTL14, WTAP, m6A eraser ALKBH5, and m6A readers YTHDF1, YTHDF2, YTHDF3 and IGF2BP1, have been shown their important roles in the ovarian and other cancers^13,22,38,39^. In an m6A-dependent way, IGF2BP1 inhibited the miRNA-mediated degradation and thus increased the expression of SRF, FOXK1, and PDLIM7^40,41^. Increased expression of IGF2BP1 is associated with a poor prognosis of ovarian and other cancers^40–42^. METTL3 was shown to promote ovarian cancer cell proliferation, invasion, and migration through targeting pri-miRNA 126-5p, lncRNA RHPN1 antisense RNA 1 (head to head) (RHPN1-AS1) and AXL receptor tyrosine kinase (AXL) mRNA^18,43,44^. ALKBH5 erased the m6A modification of Janus kinase 2 (JAK2) and stabilized JAK2 mRNA, leading to cisplatin resistance in ovarian cancer^45^. The m6A reader YTHDF1 promoted ovarian cancer progression via augmenting EIF3C translation^46^. In addition, data from the TCGA database showed the differential expression of tIGF2BP1, YTHDF1 and YTHDF2 genes between normal and ovarian cancer tissues. This study did not observe the difference in expression of these genes between HGSOC and normal FT tissues. Though more studies are needed to corroborate the result, our study implies that FTO, IGF2BP2 and IGF2BP3 may play more important roles than other regulators in the m6A regulation in HGSOC in Chinese women. It is worthwhile to further study mechanisms in modulating these m6A regulators in ovarian cancer.

GO analysis revealed that the G protein-coupled receptor (GPCR) signaling pathway was one of the functions related to m6A modification. GPCRs are the largest family of cell membrane receptors involving signal transduction related to many key physiological processes. Recent studies have shown that the aberrant GPCRs contribute to many aspects of tumorigenesis, including proliferation, invasion, angiogenesis, metastasis, and survival^47,48^. Systematic analysis of large-scale genomes revealed that GPCRs were mutated in about 20% of all human tumors, including ovarian cancer^49^. The alterations of gene expression and promoter methylation of GPCRs have also been reported previously^50^. An estrogen receptor GPER, a member of the GPCR family, was reported to promote the migration and invasion of ovarian cancer cells^51^. In contrast, other studies suggested that GPER was correlated with the prolonged overall survival of epithelial ovarian cancer^52,53^, and the agonist of GPER can inhibit ovarian cancer cell proliferation^54^.

KEGG analysis showed that m6A hyper-methylated genes were enriched in the FoxO signal pathway. The forkhead box O (FoxO) family of transcriptional factors consists of four members: FoxO1, FoxO3, FoxO4 and FoxO6. They participate in the regulation of various steps of cancer initiation and metastasis, though their regulation patterns have not been fully illustrated. Sisci et al.^55^ reported that in breast cancer, FoxO3a overexpression decreased the motility, invasiveness, and anchorage-independent growth in estrogen receptor α-positive (ERα+) cells, while played an opposite role in ERα-silenced cells and in ERα-negative (ERα−) cell lines. Higher expression of FOXO1/PAX3 was associated with poor prognosis in epithelial ovarian cancer^56^. Xie et al.^57^ found that FOXO1 repression contributed to cHL lymphomagenesis. FOXO1 was negative in almost all classical Hodgkin lymphoma (cHL) cases and had low expression in cHL cell lines. Ectopic expression of FOXO1 induced apoptosis in cHL cells and blocked proliferation by arresting the cell-cycle in the G0/G1 phase. FOXO3a promoted invasive migration by inducing the expression of matrix metalloproteinase 9 (MMP-9) and MMP-13, whereas depletion of FOXO3a in breast cancer cells leads to decreased tumor size specifically due to attenuated invasive migration^58^. Together with our data, these findings support the m6A modulation of the FoxO family in the development and progression of ovarian cancer.

This study also identified many other differentially m6A methylated and expressed genes involving signaling pathways related to ovarian pathogenesis and progression^37^. Our data showed that the Wnt singling pathway is one of the m6A modulated pathways in HGSOC. The previous study indicated that upregulation of the Wnt signaling pathway by elevated m6A contributed to Poly (ADP-ribose) polymerase inhibitors (PARPi) resistance. The Wnt signaling pathway is a potential therapeutic target in epithelial ovarian cancer^35^. All m6A modulated genes and their related complex signaling pathways broaden our understanding of the molecular mechanism for pathogenesis, drug resistance and progression of ovarian cancer. More studies are warranted to utilize these genes and pathways as biomarkers for early diagnosis and targets for treatment.

Limitations of this study include that both MeRIP-seq and RNA-seq were conducted in a small number of samples. As a retrospective cross-sectional study, there is a potential bias in sample selection. This study is limited to examining profiling data at one time point. The changes in gene methylation and expression during ovarian cancer development and progression are not determined. The MeRIP-seq has its limits in the detection of m6A methylation^59^. The functions of m6A methylation regulators are fulfilled through their proteins. Their protein levels were determined with IHC staining but with a limited number of samples. The target genes are important in mediating the functions of these regulators. Further knock-out or knock-in studies are needed to reveal the functions of these regulators and their target genes in ovarian cancer development. More studies were needed to develop these identified genes as biomarkers in diagnosing HGSOC. The strength of this study is the synchronous profiling of both m6A methylation and gene expression in HGSOC and FT tissues and the validation using RT-PCR, IHC staining, and public databases.

## Conclusions

These differentially m6A methylated and expressed genes and their related signaling pathways may help to better understand molecular mechanisms for the development and progression of ovarian cancer. These genes may have the potential to diagnose and predict the prognosis of ovarian cancer. More in-depth studies are warranted to further explore their application in novel diagnosis and treatment of the disease.

### Supplementary Information

Supplementary Information.

## Data Availability

The data presented in the study are deposited in the National Genomics Data Center of China (https://ngdc.cncb.ac.cn/) under accession number of PRJCA020109.
