# Supplementary material for: Synchronous profiling of mRNA N6-methyladenosine modifications and mRNA expression in high-grade serous ovarian cancer: a pilot study
Source: Sci Rep. 2024 May 7;14:10427. doi: 10.1038/s41598-024-60975-x (PMC11076553; doi:10.1038/s41598-024-60975-x)
Supplement: Supplementary file 1 — Supplementary Information. [file 41598_2024_60975_MOESM1_ESM.docx]

**Supplementary data**

**Supplementary Tables**

**Table S1: Clinical characteristics of HGSOC patients and their controls**

| Patient ID | Age (year) | Pathological type | Treatment |
| --- | --- | --- | --- |
| 1 | 53 | HGSOC | Primary debulking |
| 2 | 53 | HGSOC | Primary debulking |
| 3 | 68 | HGSOC | Primary debulking |
| 4 | 57 | HGSOC | Primary debulking |
| 5 | 48 | HGSOC | Primary debulking |
| 6 | 58 | HGSOC | Primary debulking |
| 7 | 63 | HGSOC | Primary debulking |
| 8 | 49 | HGSOC | Primary debulking |
| 9 | 69 | HGSOC | Primary debulking |
| 10 | 45 | HGSOC | Primary debulking |
| 11 | 72 | HGSOC | Primary debulking |
| 12 | 48 | leiomyoma | Surgery |
| 13 | 57 | leiomyoma | Surgery |
| 14 | 53 | leiomyoma | Surgery |
| 15 | 53 | leiomyoma | Surgery |
| 16 | 60 | leiomyoma | Surgery |
| 17 | 58 | leiomyoma | Surgery |
| 18 | 55 | leiomyoma | Surgery |
| 19 | 49 | leiomyoma | Surgery |
| 20 | 55 | leiomyoma | Surgery |
| 21 | 52 | leiomyoma | Surgery |
| 22 | 58 | leiomyoma | Surgery |

**Table S2: QRT-PCR primer sequences used in the research**

| Gene name | Prime | Sequence |
| --- | --- | --- |
| METTL3 | Forward | TTGTCTCCAACCTTCCGTAGT |
|  | Reverse | CCAGATCAGAGAGGTGGTGTAG |
| METTL14 | Forward | GAACACAGAGCTTAAATCCCCA |
|  | Reverse | TGTCAGCTAAACCTACATCCCTG |
| ALKBH5 | Forward | GCAAGGTGAAGAGCGGCATCC |
|  | Reverse | GTCCACCGTGTGCTCGTTGTAC |
| FTO | Forward | GTTCACAACCTCGGTTTAGTTC |
|  | Reverse | CATCATCATTGTCCACATCGTC |
| WTAP | Forward | TTCCCAAGAAGGTTCGATTG |
|  | Reverse | TGCAGACTCCTGCTGTTGTT |
| YTHDF1 | Forward | CACCCAGAGAACAAAAGGACAAG |
|  | Reverse | CGGCGGGTAATAGCTGGAC |
| YTHDF2 | Forward | AGCCCCACTTCCTACCAGATG |
|  | Reverse | TGAGAACTGTTATTTCCCCATGC |
| IGF2BP1 | Forward | GGCCATCGAGAATTGTTGCAG |
|  | Reverse | CCAGGGATCAGGTGAGACTG |
| IGF2BP2 | Forward | ACACAGACACAGAAACCGCC |
|  | Reverse | AACTGATGCCCGCTTAGCTT |
| IGF2BP3 | Forward | ACGAAATATCCCGCCTCATTTAC |
|  | Reverse | GCAGTTTCCGAGTCAGTGTTCA |
| β-actin | Forward | CATGTACGTTGCTATCCAGGC |
|  | Reverse | CTCCTTAATGTCACGCACGAT |

**Table S3: Summary of reads data and quality testing of MeRIP-seq (n=3 each).**

| Sample | Raw reads | Clean reads | Valid(%) | Q30 (%) | GC(%) |
| --- | --- | --- | --- | --- | --- |
| HGSOC1_IP | 46386082 | 43435390 | 87.00 | 95.23 | 48.61 |
| HGSOC1_input | 46782876 | 45687734 | 90.97 | 95.29 | 48.19 |
| HGSOC2_IP | 46689902 | 44643944 | 88.89 | 95.34 | 49.30 |
| HGSOC2_input | 48655946 | 47678614 | 91.31 | 95.34 | 48.89 |
| HGSOC3_IP | 46606204 | 44221468 | 88.04 | 95.02 | 49.07 |
| HGSOC3_input | 46252460 | 45421490 | 91.27 | 95.34 | 48.96 |
| FT1_IP | 40837706 | 36039520 | 81.67 | 95.05 | 49.26 |
| FT1_input | 47674984 | 46411792 | 90.18 | 95.28 | 49.10 |
| FT2_IP | 40433266 | 36106094 | 82.64 | 94.89 | 49.09 |
| FT2_input | 47258212 | 46175878 | 90.58 | 95.25 | 48.57 |
| FT3_IP | 46777048 | 44061422 | 87.12 | 94.75 | 48.50 |
| FT3_input | 46843916 | 45858744 | 90.91 | 95.02 | 48.35 |

**Supplementary Figures**

**Figure S1. Overview of m6A distributions in genes between HGSOC and FT tissues (n=3 each).** (A) The percentage of the m6A peak number per gene in HGSOC and FT tissues. (B) The proportion of the m6A peak number per differentially methylated gene. FT, fallopian tube; HGSOC, high grade serous ovarian cancer.

**
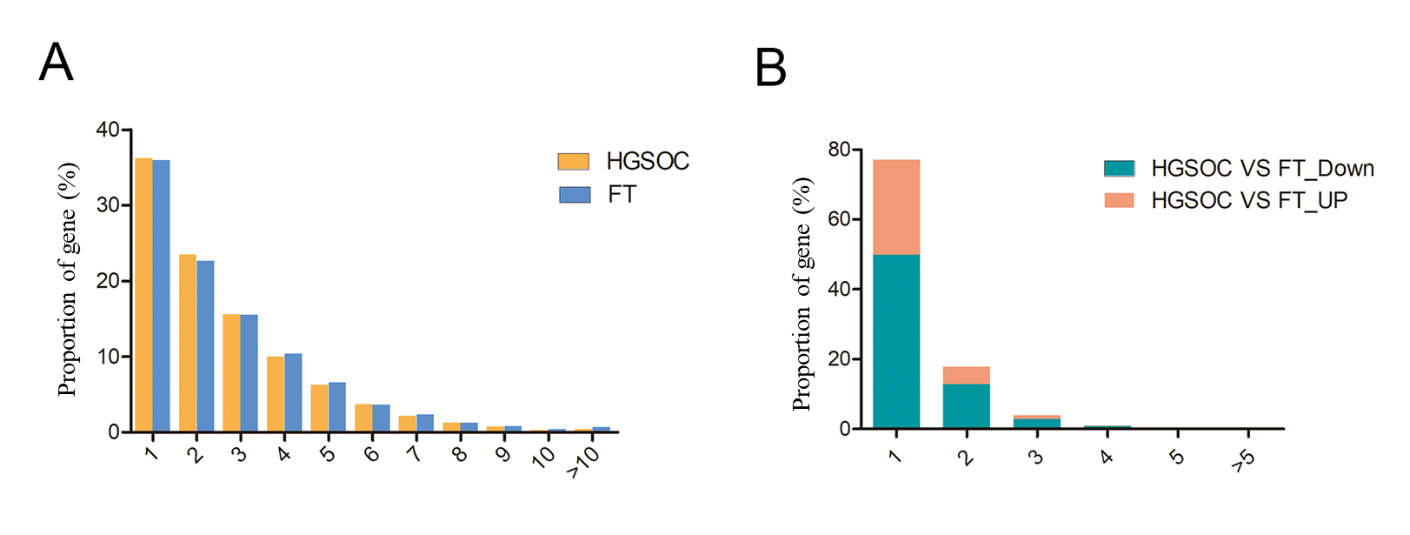
**

**Figure S2. Result of GO function and KEGG pathway analyses in four-group of differential genes by the conjoint assay.** (**A**) GO function analyses of hypermethylated and upregulated genes. (**B**) KEGG enrichment pathways of hypermethylated and upregulated genes. (**C**) GO function analyses of hypermethylated and downregulated genes. (**D**) KEGG enrichment pathways of hypermethylated and downregulated genes. (**E**) GO function analyses of hypomethylated and upregulated genes. (**F**) KEGG enrichment pathways of hypomethylated and upregulated genes. (**G**) GO function analysis of hypomethylated and downregulated genes. (**H**) KEGG enrichment pathways of hypomethylated and downregulated genes.


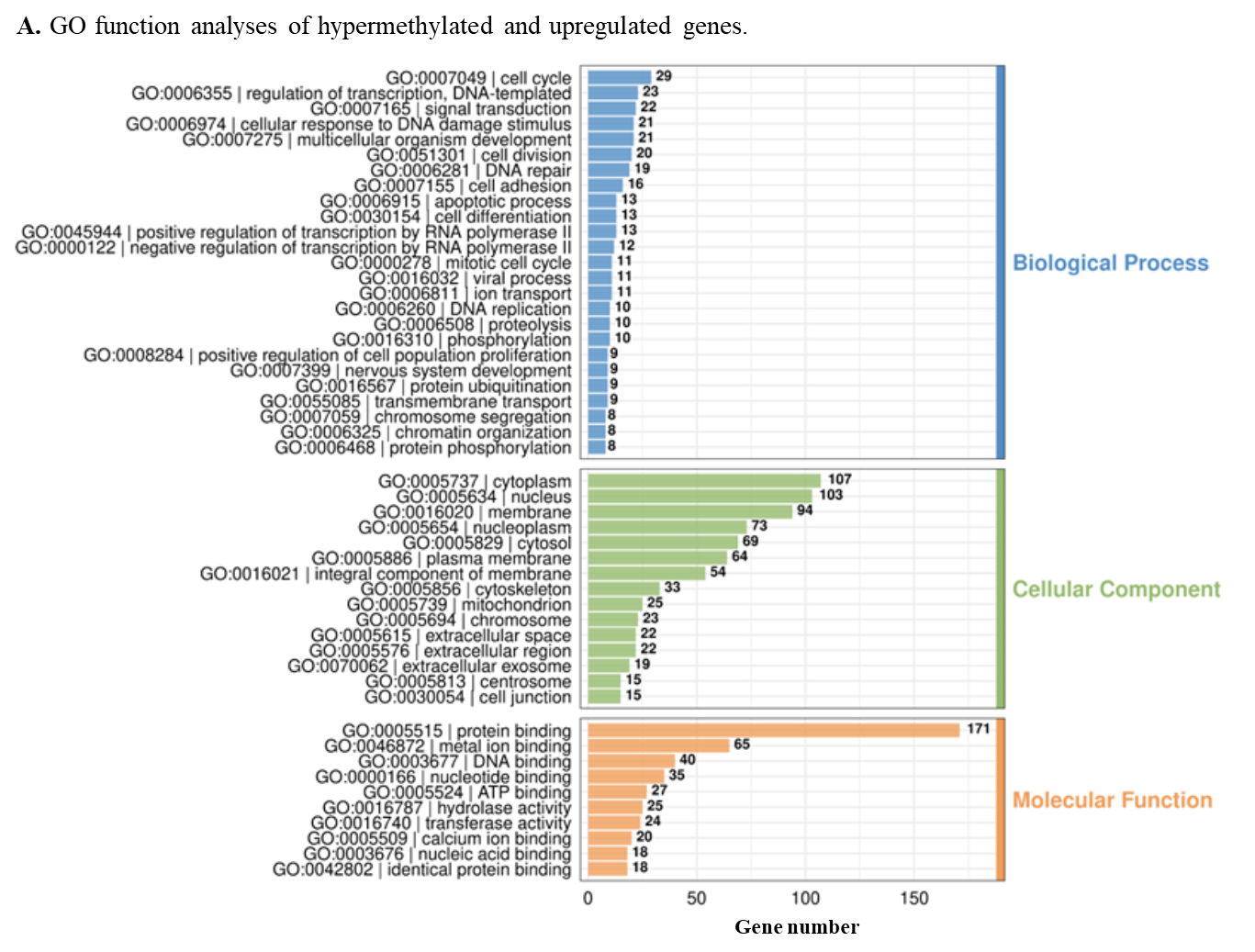


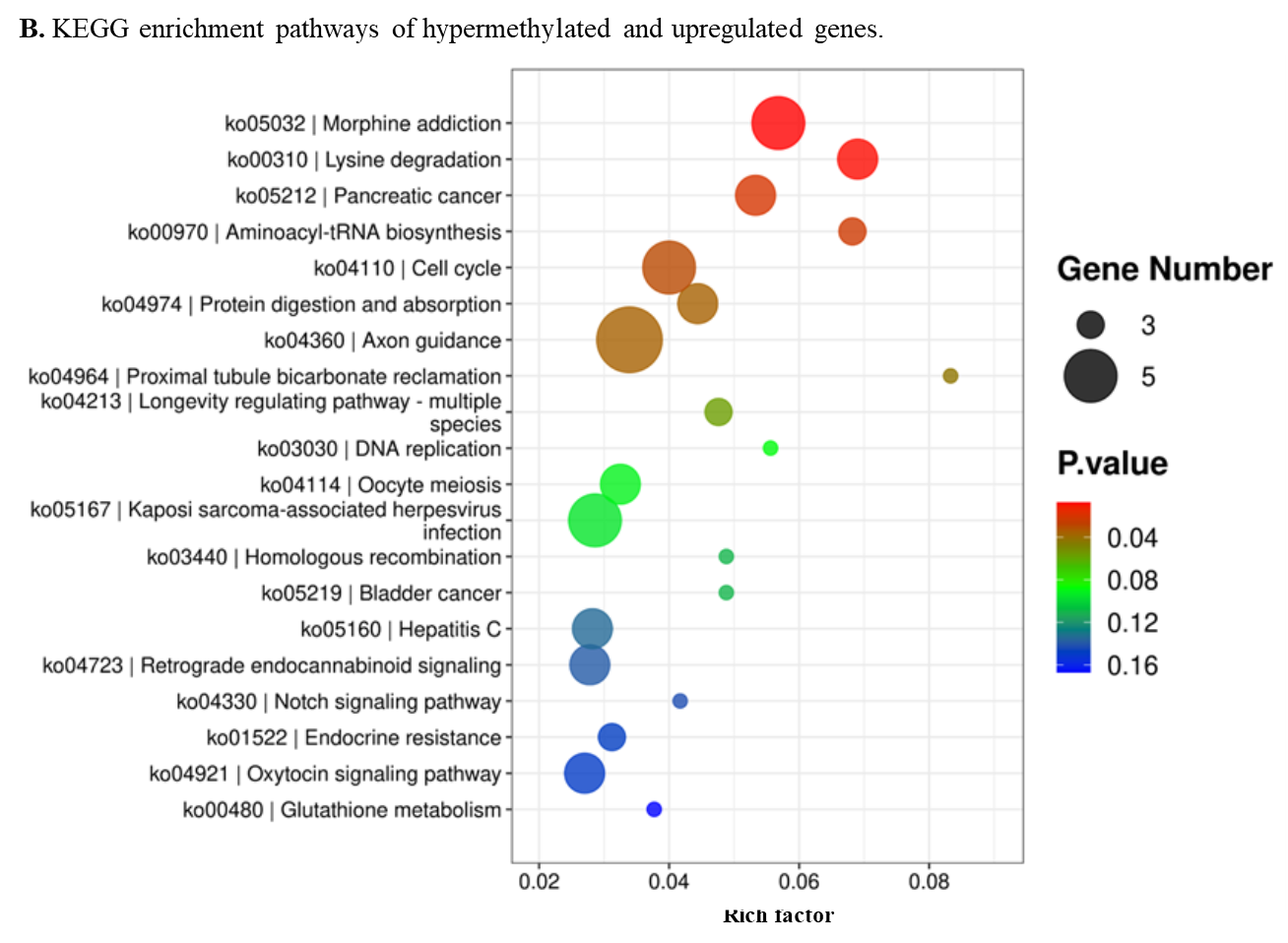


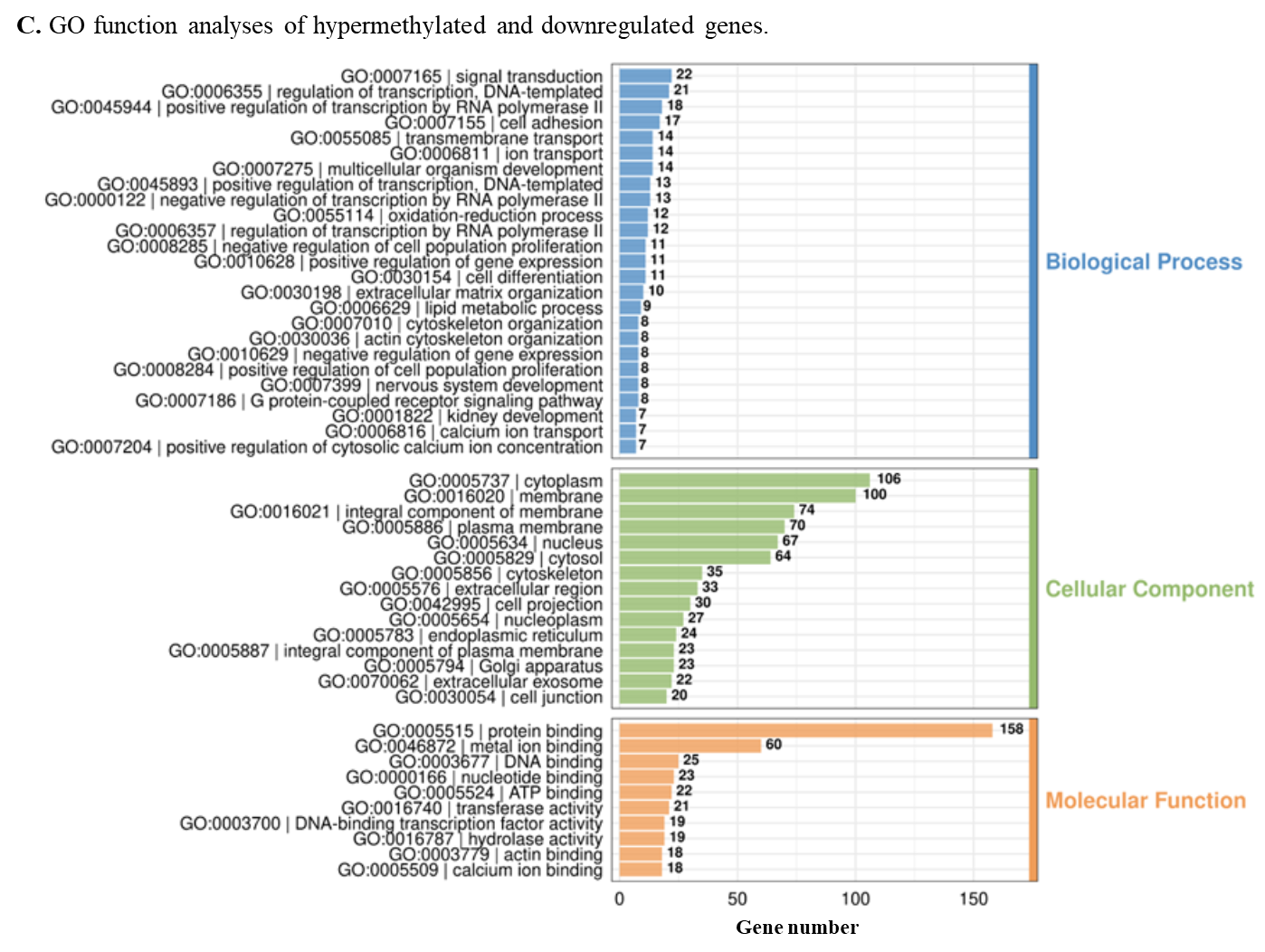


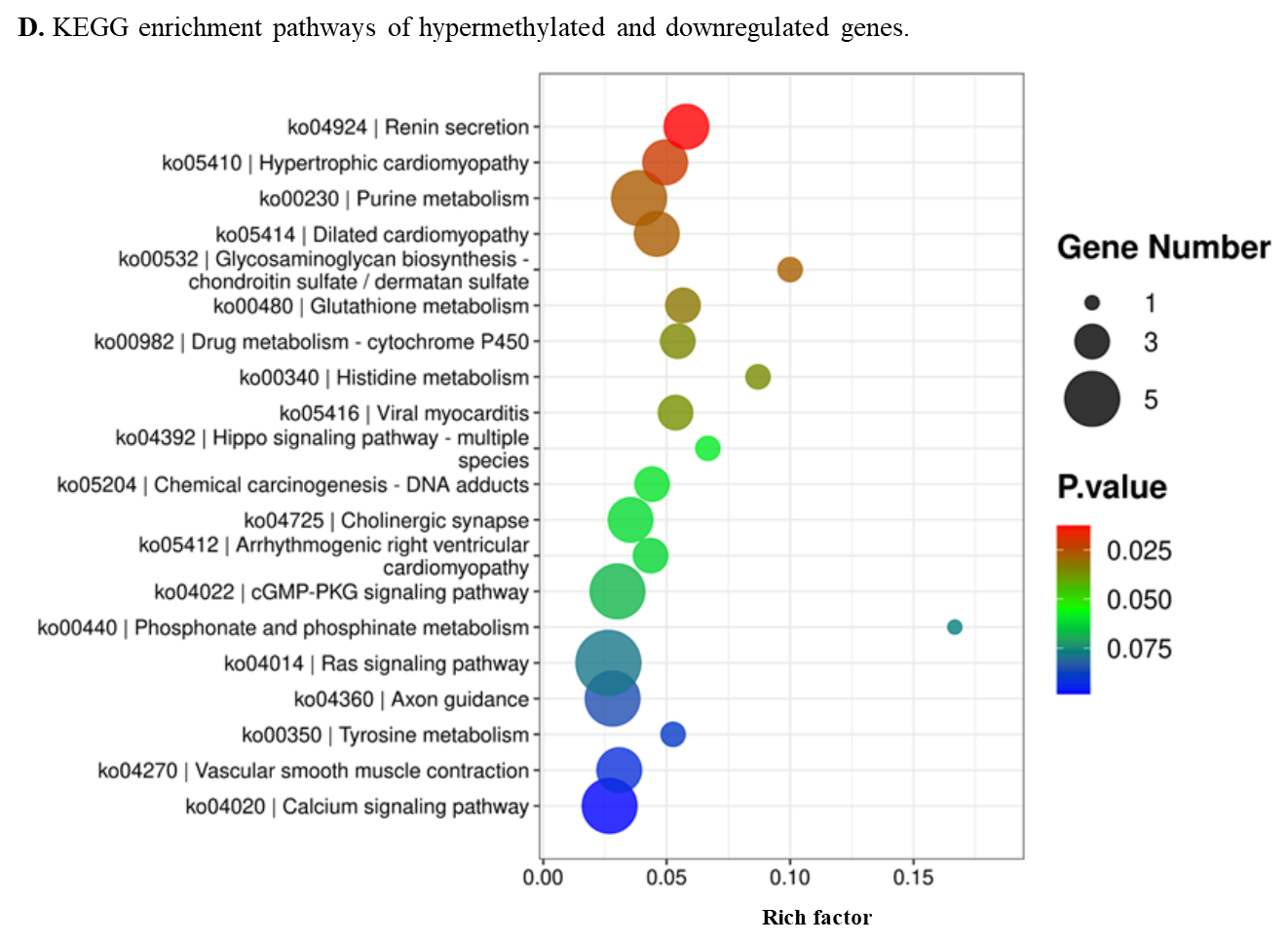


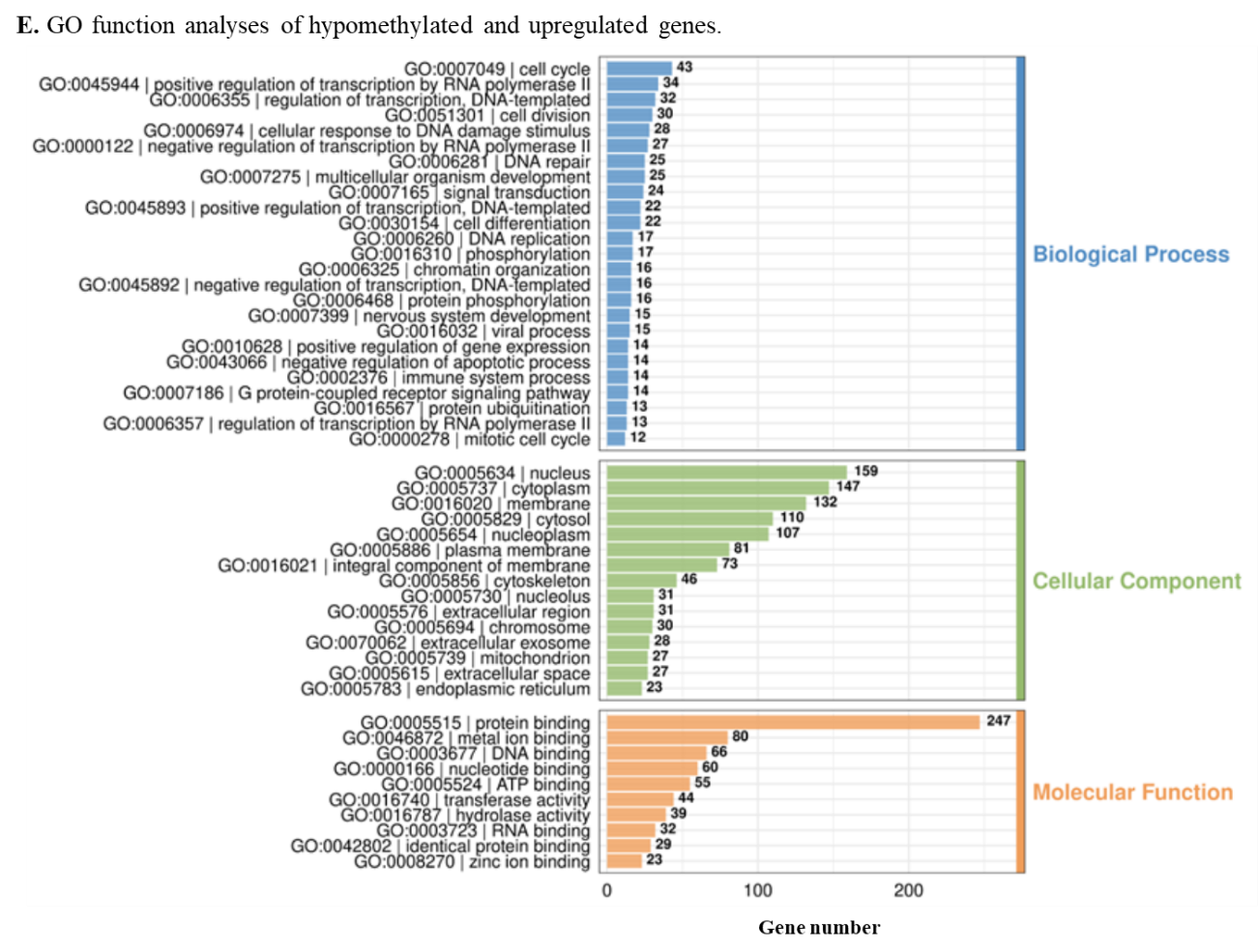


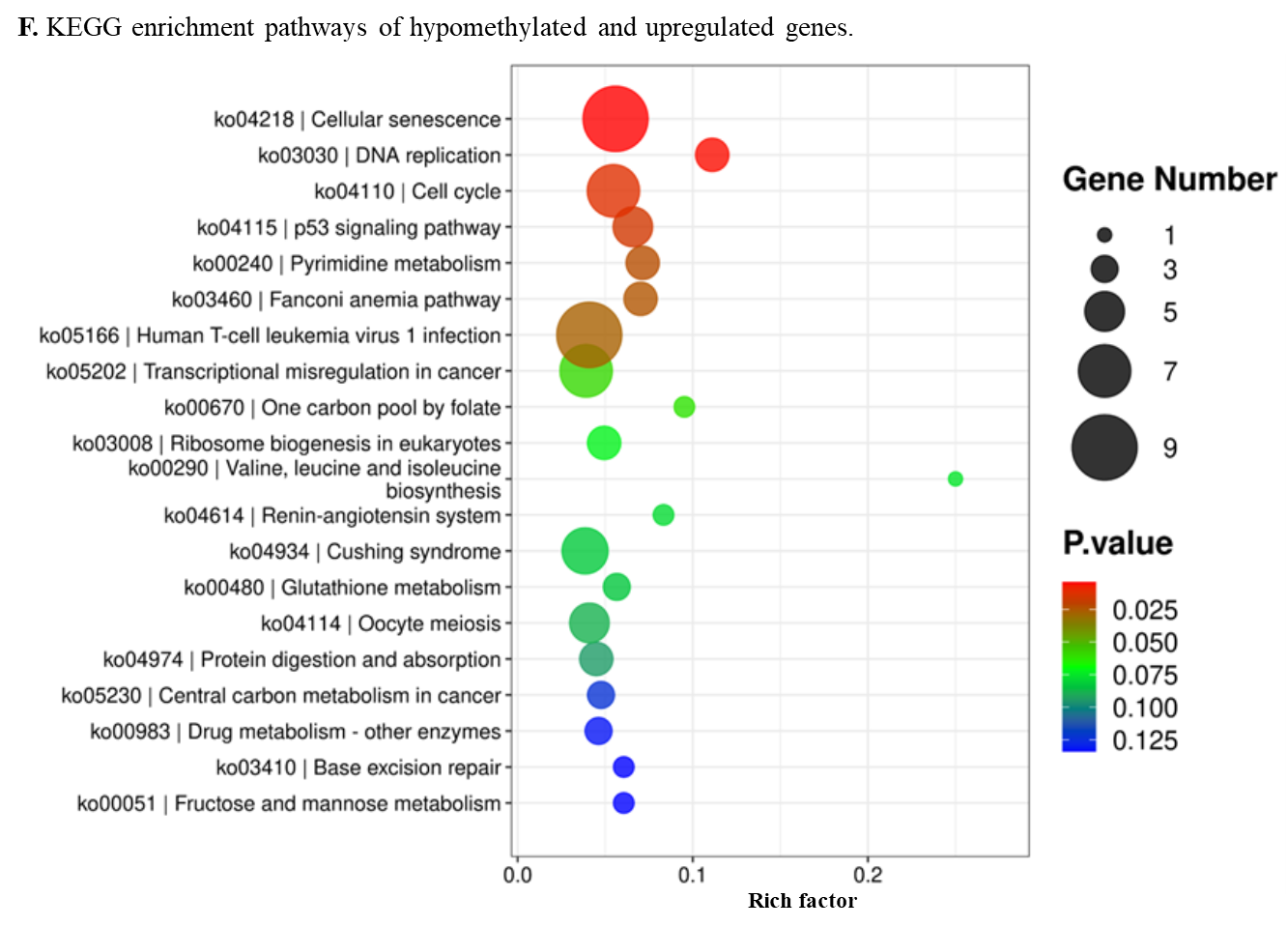


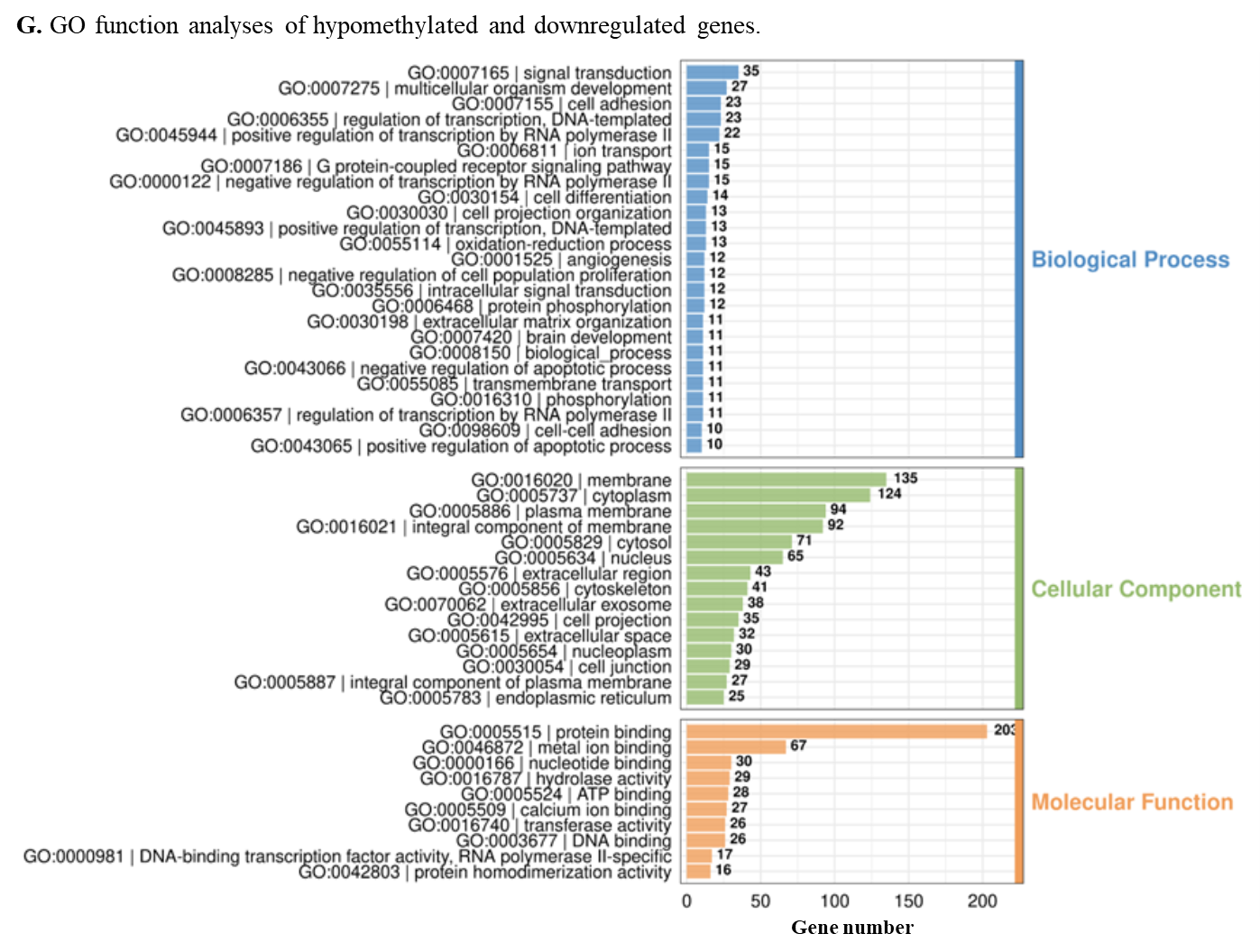


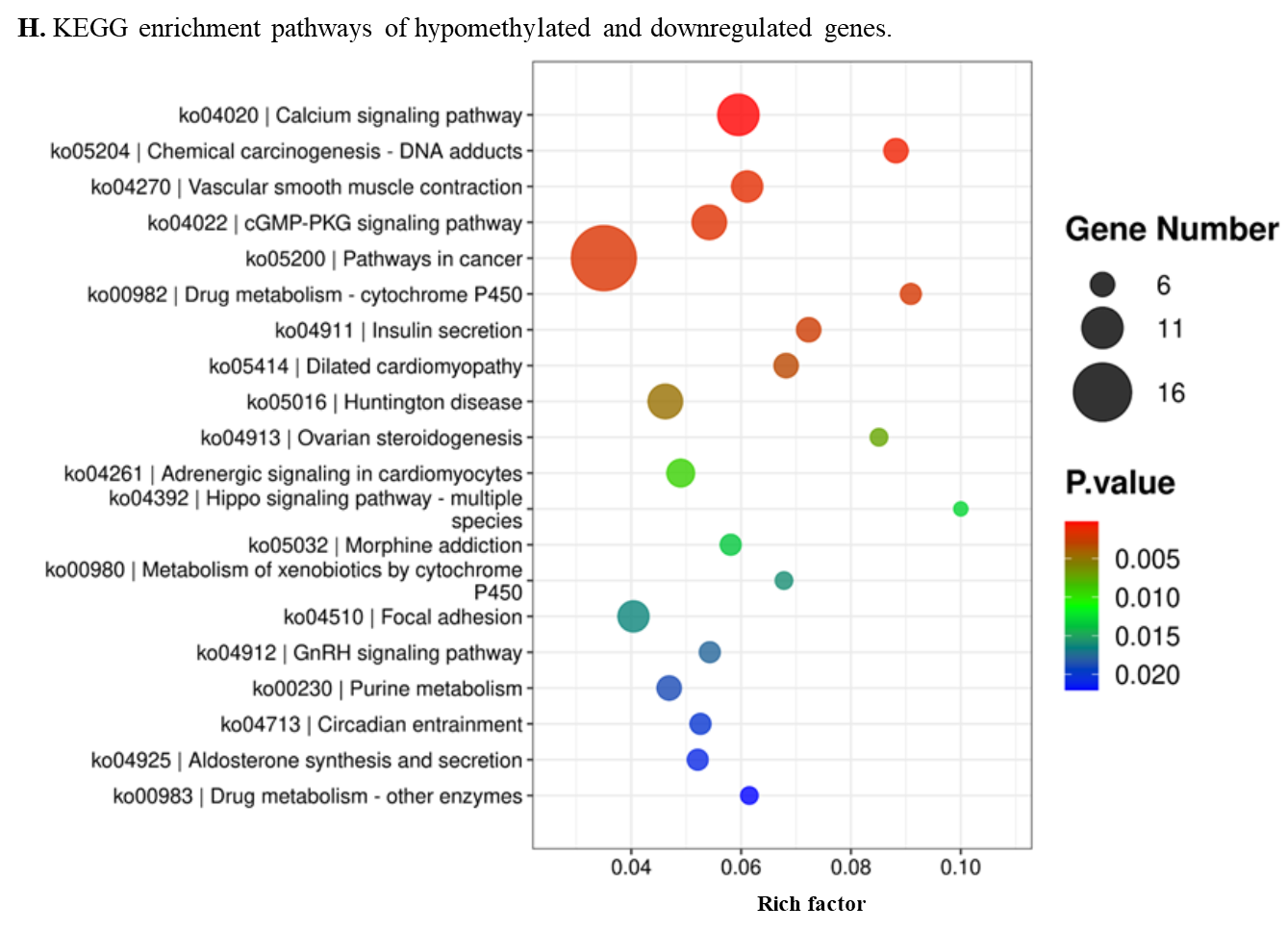


# Figure S3. Representative immunohistochemical staining of high grade serous ovarian cancer (HGSOC) (n=10) and normal fallopian tube (FT) (n=4). (A) Representative staining images for indicated antibodies. (B) Quantitative analysis of immunohistochemical images by immunoreactive scores (IRS). * indicates P value < 0.05. FT, fallopian tube; HGSOC, high grade serous ovarian cancer.

**
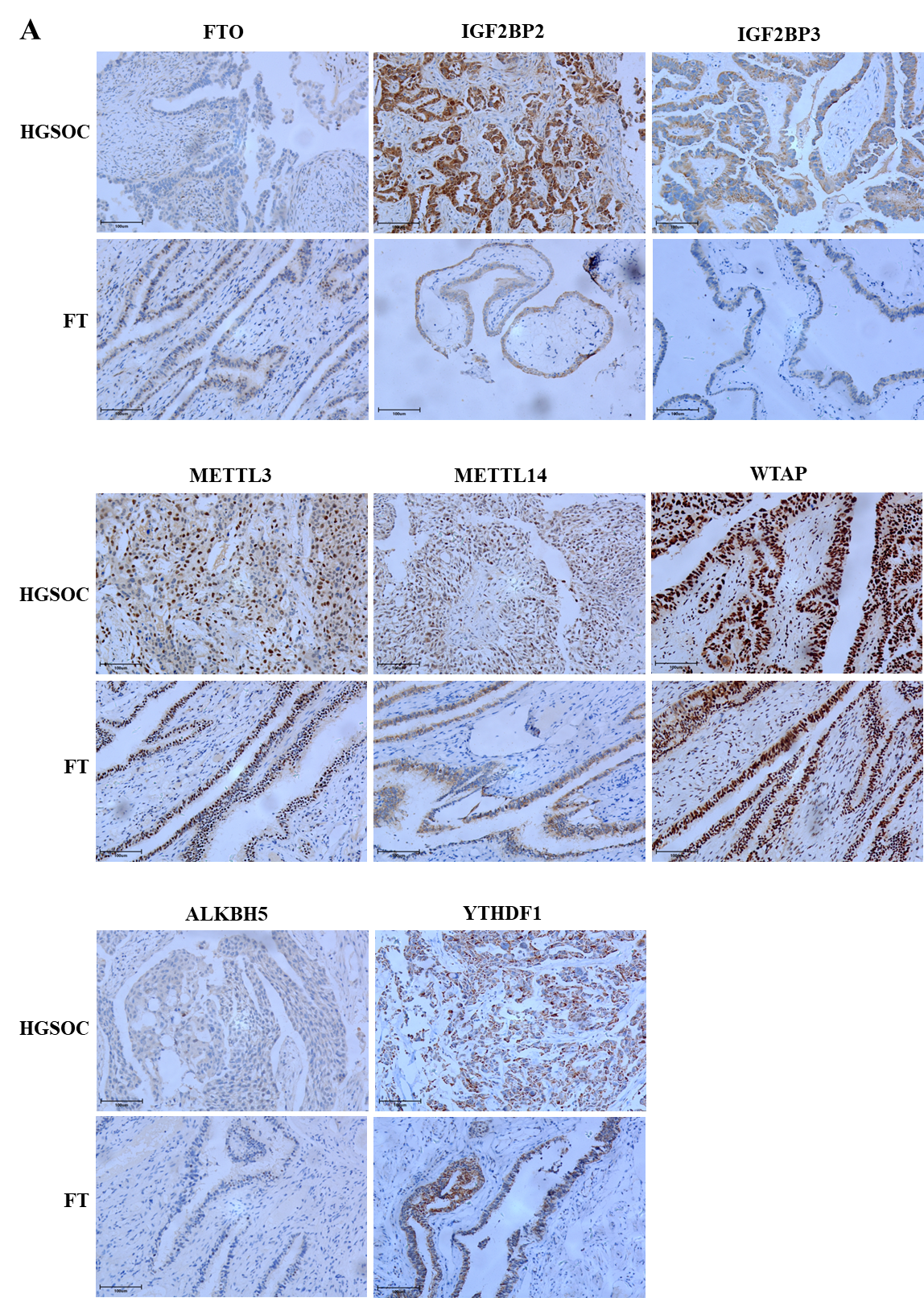
**

**
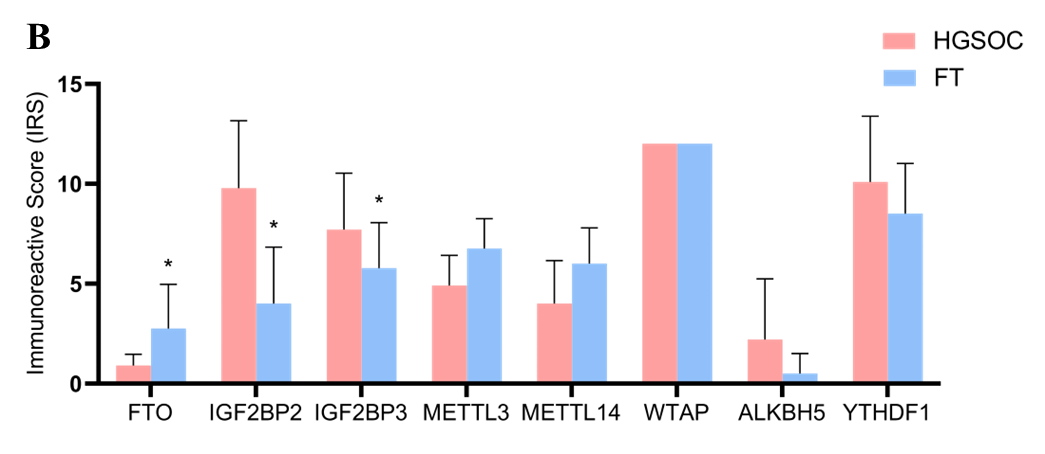
**
